# Synthesis of antibacterial 1,3-diyne-linked peptoids from an Ugi-4CR/Glaser coupling approach

**DOI:** 10.3762/bjoc.11.4

**Published:** 2015-01-07

**Authors:** Martin C N Brauer, Ricardo A W Neves Filho, Bernhard Westermann, Ramona Heinke, Ludger A Wessjohann

**Affiliations:** 1 Department of Bioorganic Chemistry, Leibniz Institute of Plant Biochemistry, Weinberg 3, D-06120 Halle/Saale, Germany

**Keywords:** antibacterial, combinatorial, diynes, homodimerization, multicomponent reactions, peptoids, Ugi reaction

## Abstract

A library of ten 1,3-diyne-linked peptoids has been synthesized through an Ugi four-component reaction (U-4CR) followed by a copper-catalysed alkyne homocoupling (Glaser reaction). The short and chemoselective reaction sequence allows generating diverse (pseudo) dimeric peptoids. A combinatorial version allows the one-pot preparation of, e.g., six-compound-libraries of homo- and heterodimers verified by ESI-MS and HPLC. In a preliminary evaluation, some compounds display moderate activity against the Gram-positive bacterium *Bacillus subtilis*.

## Introduction

**Figure 1:**
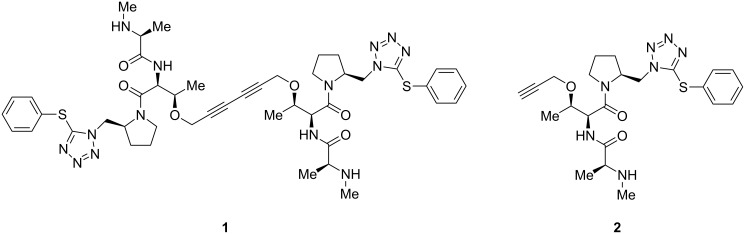
Apoptosis inducer *C*_2_-symmetric 1,3-diyne-linked peptide **1** and its inactive monomer **2**.

A re-occurring principle of nature to mediate or increase biological activity is dimerization [1]. Many protein receptors dimerize upon activation and recruit their active form by this transformation. This process is mainly initiated by dimeric natural products or symmetric bivalent ligands, which can be of peptidic origin [2–3]. As an example, Harran and co-workers synthesized a low-molecular weight *C*_2_-symmetric 1,3-diyne-linked peptide **1** which was able to mimic the function of Smac (second mitochondria-derived activator of caspase) protein by triggering caspase 8 activation as well as apoptosis at concentrations as low as 100 pM. The higher activity of **1** in comparison to **2** (Figure 1) is possibly related to the ability of **1** to interact simultaneously with adjacent baculovirus inhibitory repeat (Bir) domains in the human X chromosome that encodes IAP (inhibitor of apoptosis) [4]. In another study Chen and co-workers found a GLP-1R antagonist only because of an unexpected dimerization [5]; and a dimer of *S*-adenosylmethionine is up to 13-fold more active than the monomer for promoting the binding of *Escherichia coli* methionine repressor to its operator DNA [6].

Peptoids are compounds which are able to mimic peptide structures [7–9]. In addition to the mimetic function, these compounds also possess an enhanced resistance to proteolytic enzymes. The fastest method for synthesizing peptoids is the Ugi four-component reaction (U-4CR) [10–12]. In combination with other protocols, this reaction has been used in the synthesis of bioactive peptides and pseudopeptides, e.g., tubulysin mimetics [13], julocrotine derivatives [14], architecturally complex peptoid macrocycles [15–16], building blocks for diversity-oriented synthesis [17], and heterocyclic compounds [18]. Complex structures as well as simple Ugi products exhibit promising biological profiles, e.g., cytotoxicity [13,19–20], fungicidal [21–22] and antibacterial properties [23–26], or inhibition of histone deacetylases [27]. The Ugi post-modification strategy has also been employed in the synthesis of heterocyclic and natural product inspired compounds [28–32]. Although several protocols of U-4CR followed by transition metal-catalysed reactions have been published so far [33], to the best of our knowledge, there are no reports about U-4CR/Glaser-type (homo) coupling combinations.

In view of an increasing interest to synthesize dimerized peptidomimetics with pharmacological properties through a step-efficient protocol that allows rapid access to highly diverse dimer libraries, we set out to develop a strategy based on an U-4CR/Glaser-type homocoupling sequence [34]. In comparison to popular cross linking reactions like, e.g., click reactions or amide bonds, the Glaser coupling allows the use of truly identical monomers. This decreases the number of steps for appropriate starting materials, and allows access to true homodimers in sensu strictu.

## Results and Discussion

To achieve the synthesis of monomers eligible for dimerizations by Glaser coupling, equimolar amounts of propargylamine (**3**), aldehyde **4**, carboxylic acid **5**, and isocyanides **6** were reacted in methanol at room temperature over 24 h following well established Ugi protocols [12]. After flash column chromatography *N*-propargyl peptoids **7a–j** were obtained in good yields. The next step was the copper-catalysed homocoupling (Glaser reaction) of the terminal alkyne functions. Albeit several protocols are reported for this reaction, the CuCl-catalyzed method recently described by Jia and co-workers was utilised to access the *C*_2_-symmetric 1,3-diynes because it does not require expensive catalysts, ligands, or additives (Table 1) [34]. The coupling reaction was clean without notable side product formation as confirmed by TLC analysis, and the desired peptoid dimers **8a–j** could be obtained in high to quantitative yields. Aromatic as well as aliphatic carboxylic acids and aldehydes have been successfully employed in both multicomponent and coupling reactions. When performing the reaction with methyl isocyanoacetate (Table 1, entry 2) the desired products could be obtained in good yields with the ester group remaining untouched. It is important to note that different protecting groups can be used: Boc-, PhAc- and Cbz-protected peptoid derivatives (Table 1, entries 8–10) reacted to the corresponding dimers **7h–j** without complications. The structure of the compounds **7a–j**, as well as **8a–j**, have been confirmed by ^1^H, ^13^C NMR spectra, and HRMS. In addition, HPLC analyses revealed that an adjacent stereocenter (Table 1, entry 8, **7h/8h**) does not racemize under the reaction conditions of both the MCR and the Glaser coupling.

**Table 1:** Synthesis of compounds **7a–j** and **8a–j**.

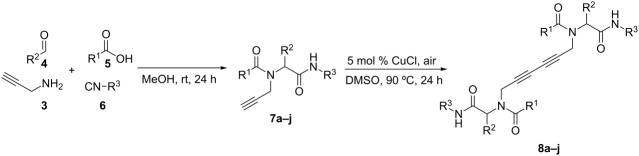					

Entry	R^1^	R^2^	R^3^	Monomer **7** yield (%)	Dimer **8** yield (%)

1	CH_3_	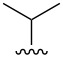	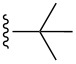	**7a** 97	**8a** 88
2	CH_3_	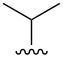	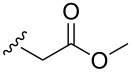	**7b** 95	**8b** 80
3	CH_3_	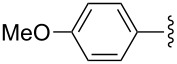	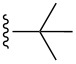	**7c** 99	**8c** 99
4	Ph	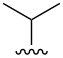	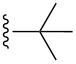	**7d** 70	**8d** 91
5	*n*-C_3_H_7_	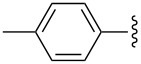	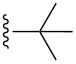	**7e** 91	**8e** 99
6	*n*-C_3_H_7_	H	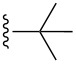	**7f** 70	**8f** 99
7	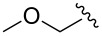	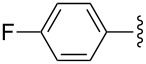	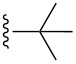	**7g** 98	**8g** 97
8	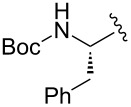	H	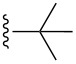	**7h** 82	**8h** 99
9	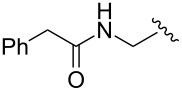	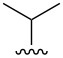	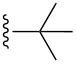	**7i** 82	**8i** 96
10	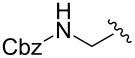	H	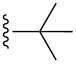	**7j** 80	**8j** 99

**Scheme 1:**
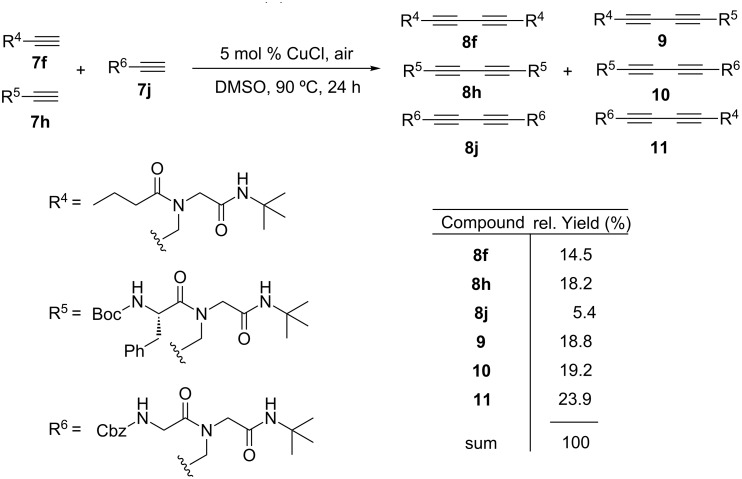
Combinatorial Glaser coupling involving acetylenes **7f**, **7j** and **7h**.

Due to the high selectivity and high conversions found in the Glaser coupling step, our attention turned toward the development of a combinatorial version of the copper-catalysed homodimerization. In this strategy two or more alkyne peptoids should couple simultaneously in the same reaction vessel in order to generate small libraries of dimers. In contrast to parallel synthesis, the combinatorial approach easily generates non-symmetric dimers **9**, **10** and **11**. Thus, the peptoids **7f**, **7h** and **7j** were pooled to a Glaser reaction as depicted in Scheme 1.

**Figure 2:**
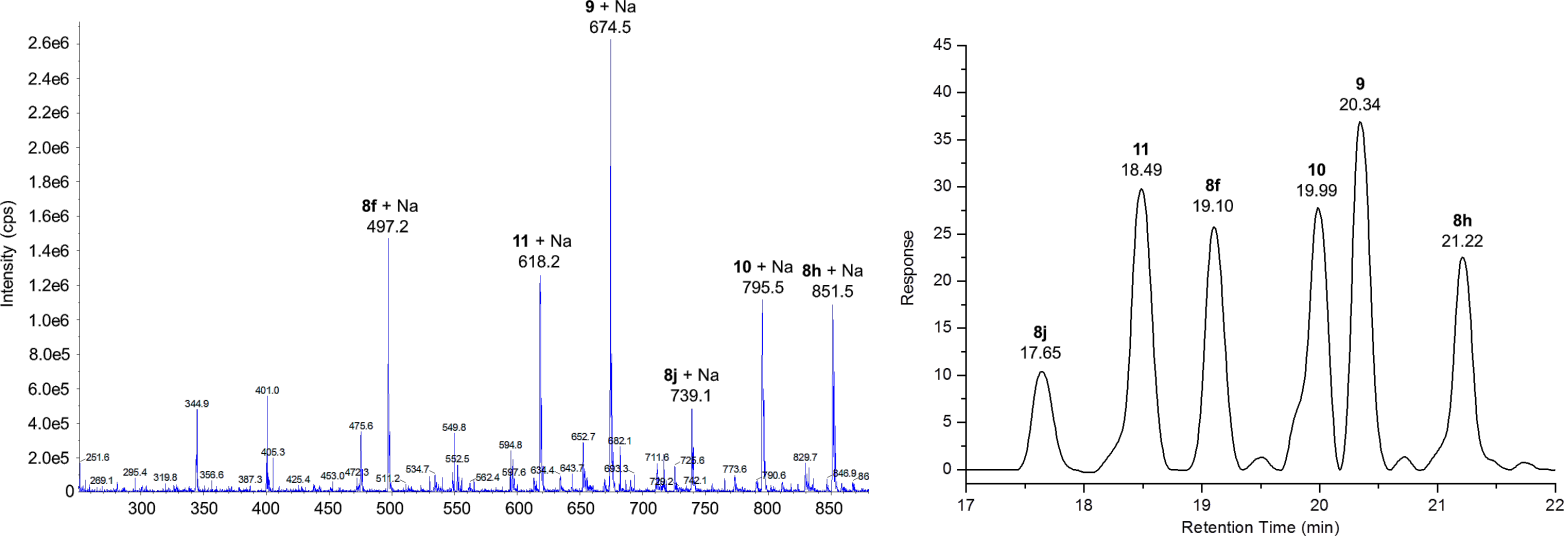
Expanded region of the ESI-MS spectrum (positive mode) and the HPLC chromatogram of the crude mixed library of 1,3-diyne peptoids (**8f**, **8h**, **8j**, **9**, **10** and **11**) produced by a combinatorial Glaser coupling of three different monomers (see Scheme 1).

The ESI-MS spectrum of the crude library confirmed the presence of all expected Glaser-coupled products **8f**, **8h**, **8j**, **9**, **10** and **11**. The HPLC–MS analysis of the composition resulted in six peaks with different retention times and intensities identified via MS as the six desired components of the library. Figure 2 illustrates the expanded region of the ESI-MS spectrum (positive mode) and the HPLC chromatogram with the respective assignments of the obtained peaks. The analysis of the obtained spectra revealed that the non-symmetric dimers **9**, **10** and **11** are formed preferentially. The abundance differences observed are mostly lower than 2-fold, in one case up to ca. 4-fold. This is still acceptable for our initial bioactivity assays, as most screening setups cover several orders of magnitude of concentration anyhow. Therefore no further attempt to optimize for an equal product distribution was deemed necessary.

**Figure 3:**
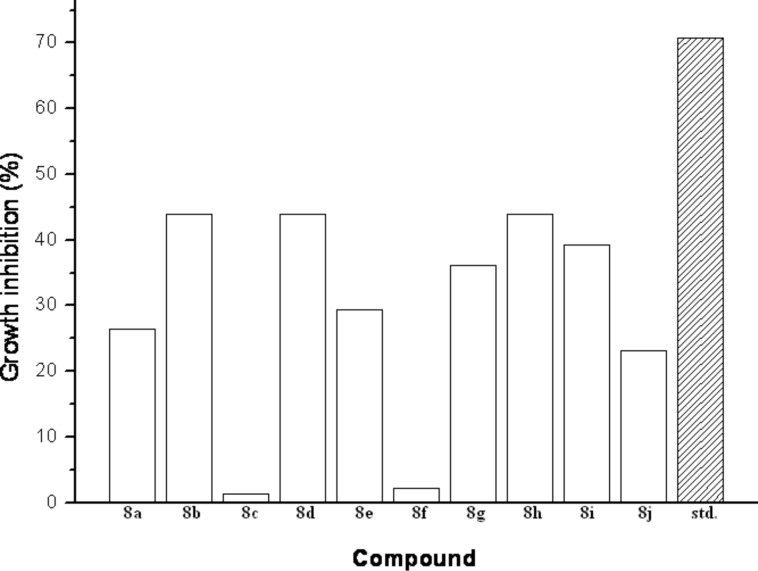
Growth inhibition of *Bacillus subtilis* by compounds **8a–j** at 1 µM (15 h), and standard erythromycin at 1 µM (15 h).

To gain insight into the antibiotic potential of the products, single compound dimers **8a–j** were subjected to a preliminary evaluation against *Bacillus subtilis* (Figure 3) [35–36]. The active compounds inhibited bacterial growth in a range from 29% to 44% at 1 μM concentration, while erythromycin as the standard led to a growth inhibition of 71% under the same assay conditions. The most active compounds were **8b**, **8d** and **8h** which displayed inhibition rates (%) of 44.0 ± 26.7, 44.0 ± 21.8 and 43.9 ± 23.0. Interestingly, compounds **8c** and **8f** showed almost no effect on bacterial growth, i.e., 1.3 ± 5.1% and 2.3 ± 13.5%, respectively, i.e., the diyne core fulfils its function as linker and spacer without itself negatively (or positively) influencing the specific activity of the active ligand moieties.

## Conclusion

In summary, a reliable sequential U-4CR/Glaser coupling approach towards the synthesis of 1,3-diyne-linked peptoids was developed. The strategy resulted in a library consisting of ten homodimers in good yields. The post-MCR copper-catalysed homocoupling reaction has also been performed in a combinatorial fashion combining three monomers. This procedure resulted in a mixed library containing six 1,3-diyne-linked symmetric and non-symmetric peptoids as confirmed by ESI-MS and HPLC experiments. Some of the synthesized compounds **8a–j** displayed growth inhibitory activity against *Bacillus subtilis* in a preliminary assay.

## Supporting Information

File 1 Complete experimental procedures, characterization and figures of ^1^H and ^13^C NMR spectra.

## References

[1] Marianayagan N J, Sunde M, Mathews J M (2004). Trends Biochem Sci.

[2] Hadden M K, Blagg B S J (2008). Anti-Cancer Agents Med Chem.

[3] Lian G, Yu B (2010). Chem Biodiversity.

[4] Li L, Thomas R M, Suzuki H, De Brabander J K, Wang X, Harran P G (2004). Science.

[5] Chen D, Liao J, Li N, Zhou C, Liu Q, Wang G, Zhang R, Zhang S, Lin L, Chen K (2007). Proc Natl Acad Sci U S A.

[6] Joce C, White R, Stockley P G, Warriner S, Turnbull W B, Nelson A (2012). Bioorg Med Chem Lett.

[7] Seo J, Lee B-C, Zuckermann R N (2011). Comprehensive Biomaterials.

[8] Vagner J, Qu H, Hruby V J (2008). Curr Opin Chem Biol.

[9] Zuckermann R N, Kerr J M, Kent S B H, Moos W H (1992). J Am Chem Soc.

[10] Zhu J, Bienaymé H (2005). Multicomponent Reactions.

[11] Vercillo O E, Andrade C K L, Wessjohann L A (2008). Org Lett.

[12] Wessjohann L A, Kaluderovic G, Neves Filho R A W, Morejon M C, Lemanski G, Ziegler T, Müller T J J (2013). Multicomponent Reactions 1: Further Components Carboxylic Acids and Amine (Ugi Reaction). Science of Synthesis.

[13] Pando O, Stark S, Denkert A, Porzel A, Preusentanz R, Wessjohann L A (2011). J Am Chem Soc.

[14] Neves Filho R A W, Westermann B, Wessjohann L A (2011). Beilstein J Org Chem.

[15] Rivera D G, Wessjohann L A (2007). Molecules.

[16] Rivera D G, Wessjohann L A (2009). J Am Chem Soc.

[17] Neves Filho R A W, Stark S, Morejon M C, Westermann B, Wessjohann L A (2012). Tetrahedron Lett.

[18] Akritopoulou-Zanze I, Djuric S W (2007). Heterocycles.

[19] Yamada R, Cao X, Butkevich A N, Millard M, Odde S, Mordwinkin N, Gundla R, Zandi E, Louie S G, Petasis N A (2011). J Med Chem.

[20] Olsen C A, Ziegler H L, Nielsen H M, Frimodt-Møller N, Jaroszewski J W, Franzyk H (2010). ChemBioChem.

[21] Galetti M D, Cirigliano A M, Cabrera G M, Ramírez J A (2012). Mol Diversity.

[22] Lamberth C, Jeanguenat A, Cederbaum F, De Mesmaeker A, Zeller M, Kempf H-J, Zeun R (2008). Bioorg Med Chem.

[23] Socha A M, Tan N Y, LaPlante K L, Sello J K (2010). Bioorg Med Chem.

[24] Neves Filho R A W, Stark S, Westermann B, Wessjohann L A (2012). Beilstein J Org Chem.

[25] Kodadek T (2013). Chem Biol.

[26] Hu Y, Amin M N, Padhee S, Wang R E, Qiao Q, Bai G, Li Y, Mathew A, Cao C, Cai J (2012). ACS Med Chem Lett.

[27] Grolla A A, Podestà V, Chini M G, Di Micco S, Vallario A, Genazzani A A, Canonico P L, Bifulco G, Tron G C, Sorba G (2009). J Med Chem.

[28] Keating T A, Armstrong R W (1996). J Am Chem Soc.

[29] Hulme C, Gore V (2003). Curr Med Chem.

[30] Hulme C, Morrissette M M, Volz F A, Burns C J (1998). Tetrahedron Lett.

[31] Wessjohann L A, Rhoden C R B, Rivera D G, Vercillo O E (2010). Top Heterocycl Chem.

[32] Wessjohann L A, Ruijter E (2005). Top Curr Chem.

[33] Koopmanschap G, Ruijter E, Orru R V A (2014). Beilstein J Org Chem.

[34] Yin K, Li C, Li J, Jia X (2011). Green Chem.

[35] Heinke R, Franke K, Porzel A, Wessjohann L A, Ali N A A, Schmidt J (2011). Phytochemistry.

[36] Michels K, Heinke R, Kuipers O P, Arnold N, Wessjohann L A J Antibiot.

