# Supplementary material for: Synthesis of antibacterial 1,3-diyne-linked peptoids from an Ugi-4CR/Glaser coupling approach
Source: Beilstein J Org Chem. 2015 Jan 7;11:25–30. doi: 10.3762/bjoc.11.4 (PMC4311588; doi:10.3762/bjoc.11.4)
Supplement: File 1 — Complete experimental procedures, characterization and figures of 1H and 13C NMR spectra. [file Beilstein_J_Org_Chem-11-25-s001.pdf]

Supporting Information

for

Synthesis of antibacterial 1,3-diyne-linked peptoids from an Ugi-4CR/Glaser coupling approach

Martin C. N. Brauer, Ricardo A. W. Neves Filho, Bernhard Westermann, Ramona Heinke and Ludger A. Wessjohann*,§

Address: Department of Bioorganic Chemistry, Leibniz Institute of Plant

Biochemistry, Weinberg 3, D-06120 Halle/Saale, Germany

* Corresponding author

Email: Ludger A. Wessjohann - wessjohann@ipb-halle.de

§Fax: +49345 5582 1309; Tel: +49 345 5582 1301

Complete experimental procedures, characterization and figures of ^{1}H and ^{13}C NMR spectra

Experimental part

General

All commercially available chemicals were used without further purification. ^{1}H NMR ($CDCl_3$, 400 MHz) and ^{13}C NMR ($CDCl_3$, 100 MHz) spectra were recorded in $CDCl_3$ solutions on a Varian Mercury 400 spectrometer at 400 (^{1}H) and 100 MHz (^{13}C), respectively. Chemical shifts (δ) are reported in ppm relative to TMS (^{1}H NMR) and to residual $CDCl_3$ signal (^{13}C NMR). High resolution ESI mass spectra were obtained from a Bruker Apex III Fourier transform ion cyclotron resonance (FT–ICR) mass spectrometer equipped with an Infinity™ cell, a 7.0 Tesla superconducting magnet, an RF-only hexapole ion guide and an external electrospray ion source (Agilent, off axis spray). ESI-MS was recorded on a Finnigan TSQ 7000, LC-Tech Ultra Plus pumps, Linear UV–vis 200 detector, Sepserve Ultrasep ES RP-18 5 µm 1 × 100 mm column, flow 70 µL min^{-1}. Flash column chromatography was carried out using Merck silica gel 60 (0.040–0.063 mm) and analytical thin-layer chromatography (TLC) was performed using Merck silica gel 60 F254 aluminium sheets. HPLC experiments were performed in an Agilent 1100 series equipped with a column SNr. 176: YMC pack 150 × 4.6 LD 102 Å 5 µm ODS-A and UV detector (200–600 nm). The employed gradient was MeOH 0.1% formic acid: H_2O 0.1% formic acid 1 mL / min (5 µL), MeOH 2%> 20 min> 100% (5 min) at 25 °C.

General procedure for the synthesis of compounds 7a–j

To a stirred solution of aldehyde (2.5 mmol) in methanol (2.5 mL) propargylamine (0.14 g, 0.16 mL, 2.5 mmol) was added. After 30 min carboxylic acid (2.5 mmol) and isocyanide (2.5 mmol) were added. The contents were stirred for 24 h. The solvent was concentrated under reduced pressure in a rotavap. The crude material was purified by isocratic column chromatography to afford the pure product. The same solvent system used for R_f value measurements was applied for performing flash column chromatography.

N-*tert*-Butyl-3-methyl-2-(*N*-(prop-2-ynyl)acetamido)butanamide (**7a**).

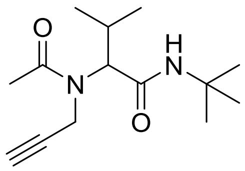

Yield: 97%. Purified by column chromatography. R_f 0.59 (EtOAc / hexane 3:7). ^{1}H NMR ($CDCl_3$, 400 MHz): δ (ppm) 6.26 (s, 1H), 4.30-4.36 (m, 2H), 3.82 and 3.85 (d, *J* = 2.4 Hz, 1H), 2.14 (t, *J* = 2.4 Hz, 1H), 2.06 (s, 3H), 2.01 (m, *J* = 4.4 Hz, 1H), 1.1.(s, 9H), 0.74 (d, *J* = 4.4 Hz, 3H), 0.69 (d, *J* = 4.4 Hz, 3H). ^{13}C NMR ($CDCl_3$, 100 MHz): δ (ppm) 171.5, 169.1, 79.2, 72.0, 62.1, 50.8, 33.7, 28.1, 26.8, 21.7, 18.9. HRMS (ESI-pos) m/z calcd for $C_{14}H_{24}N_2NaO_2$ $(M+Na)^+$ 275.1735, found 275.1729.

Methyl 2-(3-methyl-2-(*N*-(prop-2-ynyl)acetamido)butanamido)acetate (**7b**).

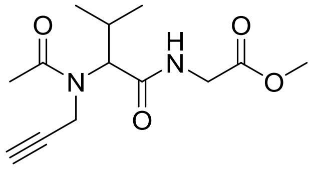

Yield: 95%. Purified by column chromatography. R_f 0.15 (EtOAc / hexane 1:1). ^{1}H NMR ($CDCl_3$, 400 MHz): δ (ppm) 7.37 (bs, 1H), 4.60 and 4.58 (s, 1H), 4.31 and 3.89 (d, *J* = 2.4 Hz, 2H), 3.73-3.88 (m, 2H), 3.57 (s, 3H), 2.24 (t, *J* = 2.4 Hz, 1H), 2.14 (m, 4H), 0.80-0.84 (m, 6H). ^{13}C NMR ($CDCl_3$, 100 MHz): δ (ppm) 172.1, 170.5, 169.7, 79.9,72.3, 61.7, 51.8, 40.5, 34.1, 26.6, 21.7, 19.1. HRMS (ESI-pos) m/z calcd for $C_{13}H_{20}N_2NaO_4$ $(M+Na)^+$ 291.1321, found 291.1315.

N-tert-Butyl-2-(4-methoxyphenyl)-2-(*N*-(prop-2-ynyl)acetamido)acetamide (**7c**).

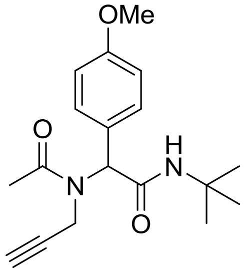

Yield: 99%. Purified by column chromatography. R_f 0.23 (EtOAc / hexane 1:1). ^{1}H NMR ($CDCl_3$, 400 MHz): δ (ppm) 7.29 (d, *J* = 8.4 Hz, 2H), 6.88 (d, *J* = 8.4 Hz, 2H), 6.15 (s, 1H), 6.00 (bs, 1H), 4.08 (d, *J* = 2.4 Hz, 2H), 3.81 (s, 3H), 2.26 (s, 3H), 2.02 (t, *J* = 2.4 Hz, 1H), 1.36 (s, 9H). ^{13}C NMR ($CDCl_3$, 100 MHz): δ (ppm) 171.5, 169.1, 159.4, 130.7, 127.0, 113.9, 79.5, 71.2, 59.7, 55.1, 51.5, 35.5, 28.5, 22.0. HRMS (ESI-pos) m/z calcd for $C_{18}H_{24}N_2NaO_3$ $(M+Na)^+$ 339.1685, found 339.1679.

N-(1-(*tert*-Butylamino)-3-methyl-1-oxobutan-2-yl)-*N*-(prop-2-ynyl)benzamide (**7d**).

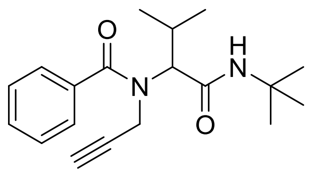

Yield: 70%. Purified by column chromatography. R_f 0.56 (EtOAc / hexane 1:4). ^{1}H NMR ($CDCl_3$, 400 MHz): δ (ppm) 7.54 (d, *J* = 6.4 Hz, 2H), 7.37-7.45 (m, 3H), 6.58 (bs, 1H), 4.21-4.30 (m, 2H), 3.87 and 3.92 (s, 1H), 2.55 (m, *J* = 6.0 Hz, 1H), 2.23 (s,1H), 1.31 (s, 9H), 0.99-1.04 (m, 6H). ^{13}C NMR ($CDCl_3$, 100 MHz): δ (ppm) 173.1, 169.0, 135.4, 130.1, 128.2, 126.8, 79.6, 72.5, 66.2, 50.9, 37.3, 28.4, 26.5, 19.5. HRMS (ESI-pos) m/z calcd for $C_{19}H_{26}N_2NaO_2$ $(M+Na)^+$ 337.1892, found 337.1886.

N-(2-(*tert*-Butylamino)-2-oxo-1-*p*-tolylethyl)-*N*-(prop-2-ynyl)butyramide (**7e**).

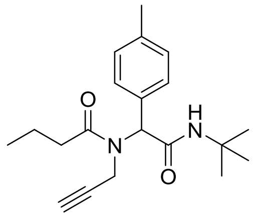

Yield: 91%. Purified by column chromatography. R_f 0.16 (EtOAc / hexane 1:4). ^{1}H NMR ($CDCl_3$, 400 MHz): δ (ppm) 7.17 (d, *J* = 8.0 Hz, 2H), 7.07 (d, *J* = 8.0 Hz, 2H), 6.07 (s, 1H), 6.06 (s, 1H), 3.98 (s, 2H), 2.41 (t, *J* = 6.4 Hz, 2H), 2.26 (s, 3H), 1.96 (s, 1H), 1.59 (m, *J* = 6.4 Hz, 2H), 1.27 (s, 9H), 0.86 (t, *J* = 6.4 Hz, 3H). ^{13}C NMR ($CDCl_3$, 100 MHz): δ (ppm) 173.4, 169.0, 137.4, 131.9, 128.9, 128.8, 79.6, 70.9, 60.1, 50.9, 34.9, 34.6, 28.1, 29.7, 17.9, 13.3. HRMS (ESI-pos) m/z calcd for $C_{20}H_{28}N_2NaO_2$ $(M+Na)^+$ 351.2048, found 351.2042.

N-(2-(*tert*-Butylamino)-2-oxoethyl)-*N*-(prop-2-ynyl)butyramide (**7f**).

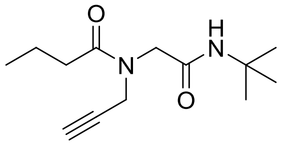

Yield: 70%. Purified by column chromatography. R_f 0.34 (EtOAc / hexane 1:1). ^{1}H NMR ($CDCl_3$, 400 MHz): δ (ppm) 6.10 and 6.06 (s, 1H), 4.29 and 4.19 (s, 2H), 3.98 (s, 2H), 2.45 and 2.25 (t, J = 6.4 Hz, 2H), 2.43 and 2.31 (t, J = 2.4 Hz, 1H), 1.68 (m, 2H), 1.37 and 1.33 (s, 9H), 0.99 (m, 3H). ^{13}C NMR ($CDCl_3$, 100 MHz): δ (ppm) 173.3, 173.1, 167.7, 166.9, 78.8, 77.8, 73.0, 72.6, 51.4, 50.9, 50.4, 38.6, 36.0, 34.7, 34.6, 28.4, 18.2, 18.0, 13.6, 13.5. HRMS (ESI-pos) m/z calcd for $C_{13}H_{22}N_2NaO_2$ $(M+Na)^+$ 261.1579, found 261.1573.

N-*tert*-Butyl-2-(4-fluorophenyl)-2-(2-methoxy-*N*-(prop-2-ynyl) acetamido) acetamide (**7g**).

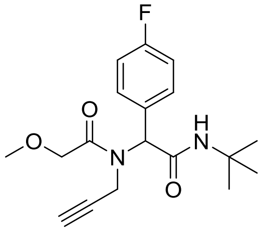

Yield: 98%. Purified by column chromatography. R_f 0.25 (EtOAc / hexane 1:1). ^{1}H NMR ($CDCl_3$, 400 MHz): δ (ppm) 7.36 (dd, J_{H-H} =6.8 Hz , J_{H-F} = 5.2 Hz, 2H), 7.05 (m, 2H), 6.20 (m, 2H), 4.30 (s, 2H), 4.19-4.06 (m, 2H), 3.41 (s, 3H), 2.07 (t, J = 2.4 Hz, 1H), 1.36 (s, 9H). ^{13}C NMR ($CDCl_3$, 100 MHz): δ (ppm) 169.9, 168.1, 164.0, 160.8, 131.2, 131.0, 130.4, 130.3, 115.6, 115.3, 78.6, 71.8, 70.8, 59.0, 58.9, 51.5, 33.7, 28.3. HRMS (ESI-pos) m/z calcd for $C_{18}H_{23}FN_2NaO_3$ $(M+Na)^+$ 357.1590, found 357.1585.

(S)-*tert*-Butyl 1-((2-(*tert*-butylamino)-2-oxoethyl)(prop-2-ynyl)amino)-1-oxo-3-phenylpropan-2-ylcarbamate (**7h**).

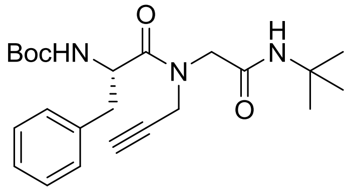

Yield: 82%. Purified by column chromatography. R_f 0.34 (EtOAc / hexane 3:7). ^{1}H NMR ($CDCl_3$, 400 MHz): δ (ppm) 7.09-7.18 (m, 5H), 6.28 and 6.87 (s, 1H), 5.55 and 5.80 (d, J = 8.0 Hz, 1H), 4.47 and 4.72 (q, J = 6.4 Hz, 1H), 3.58-4.35 (m, 4H), 2.77-3.07 (m, 2H), 2.17 and 2.31 (s, 1H), 1.21-1.26 (m, 18H). ^{13}C NMR ($CDCl_3$, 100 MHz): δ (ppm) 172.1, 171.9, 166.9, 166.4, 155.2, 154.9, 135.9, 135.6, 129.0, 128.1, 126.6, 126.5, 79.3, 79.2, 77.7, 77.4, 73.7, 72.5, 51.7, 51.5, 51.1, 50.8, 50.2, 49.9, 38.1, 37.8, 35.5, 28.2, 27.8. HRMS (ESI-pos) m/z calcd for $C_{23}H_{33}N_3O_4$ $(M+Na)^+$ 438.2369, found 438.2363.

N-*tert*-Butyl-3-methyl-2-(2-(2-phenylacetamido)-*N*-(prop-2-ynyl) acetamido) butanamide (**7i**).

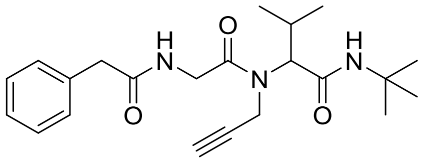

Yield: 82%. Purified by column chromatography. R_f 0.30 (EtOAc / hexane 2:3). ^{1}H NMR ($CDCl_3$, 400 MHz): δ (ppm) 7.77 and 6.77 (t, J = 4.4 Hz,1H), 7.23-7.36 (m, 5H), 6.01 (s, 1H), 4.53 and 4.05 (d, J = 2.4 Hz, 2H), 4.49-3.76 (m, 3H), 3.61 and 3.55 (s, 2H), 2.35 and 2.14 (t, J = 2.4 Hz, 1H), 2.27 (m, 1H), 1.29 and 1.27 (s, 9H), 0.94 (d, J = 6.8 Hz, 3H), 0.86 (d, J = 6.8 Hz, 3H). ^{13}C NMR ($CDCl_3$, 100 MHz): δ (ppm) 171.4, 170.6, 169.6, 168.7, 168.3, 167.8, 134.4, 134.2, 129.0, 128.9, 128.4, 126.8, 79.5, 78.2, 73.1, 70.3, 65.9, 62.9, 51.0, 42.9, 42.6, 41.0,

41.1, 32.4, 31.9, 28.0, 26.7, 19.2, 18.9. HRMS (ESI-pos) m/z calcd for $C_{22}H_{31}N_3NaO_3$ (M+Na)$^+$ 408.2263, found 408.2258.

Benzyl 2-((2-(*tert*-butylamino)-2-oxoethyl)(prop-2-ynyl)amino)-2-oxoethylcarbamate (**7j)**

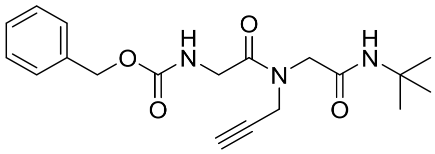

Yield: 80%. Purified by column chromatography. R_f 0.19 (EtOAc / hexane 1:1). ^{1}H NMR ($CDCl_3$, 400 MHz): δ (ppm) 7.28-7.25 (m, 5H), 6.36 and 6.16 (s, 1H), 5.95 (s, 1H), 5.04 (d, *J* = 8 Hz, 2H), 4.05 and 4.03 (s, 2H), 4.23 and 3.92 (s, 2H), 4.10 and 3.97 (s, 2H), 2.36 and 2.26 (s, 1H), 1.30 and 1.27 (s, 9H). ^{13}C NMR ($CDCl_3$, 100 MHz): δ (ppm) 169.1, 167.1, 166.2, 156.4, 136.3, 128.5, 128.1, 127.9, 78.2, 74.1, 73.3, 66.8, 51.8, 51.5, 50.3, 49.7, 42.6, 37.9, 36.4, 28.6. HRMS (ESI-pos) m/z calcd for $C_{19}H_{25}N_3NaO_4$ (M+Na)$^+$ 382.1743, found 382.1737.

General procedure for the synthesis of compounds 8a–j

In a 10 mL round bottom flask, to stirred solution of a suitable alkyne **7a–j** (0.25 mmol) in dry DMSO (0.5 mL), CuCl (1.3 mg, 0.013 mmol/5 mol %) was added. The contents were stirred at 90 °C under air atmosphere. After 24 h the reaction mixture was diluted with ethyl acetate (10 mL) and filtered through a Celite plug. The solvent was removed under reduced pressure in a rotavap. The crude material was purified by column chromatography to afford the pure product. The

same solvent system for the R_f values measurements was employed for column chromatography.

2,2'-(Hexa-2,4-diyne-1,6-diylbis(acetylazanediyl))bis(*N*-*tert*-butyl-3-methylbutanamide) (**8a**, mixture of diastereoisomers).

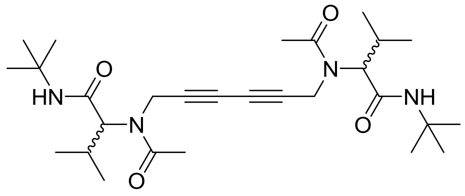

Yield: 88%. Purified by column chromatography. R_f 0.49 (EtOAc). ^{1}H NMR ($CDCl_3$, 400 MHz): δ (ppm) 5.91 (s, 4H), 4.54-4.41 (m, 8H), 4.14 and 4.09 (s, 4H), 2.24 (s, 12H), 2.18 (m, 4H), 1.31 (s, 36H), 0.94 (d, J = 6.8 Hz, 12H), 0.86 (d, J = 6.8 Hz, 12H). ^{13}C NMR ($CDCl_3$, 100 MHz): δ (ppm) 171.9, 169.1, 74.4, 68.0, 51.4, 42.6, 34.7, 28.5, 26.9, 22.0, 19.3, 19.0. HRMS (ESI-pos) m/z calcd for $C_{28}H_{46}N_4NaO_4$ $(M+Na)^+$ 525.3417, found 525.3411.

Dimethyl 6,13-diacetyl-4,15-dioxo-5,14-di(propan-2-yl)-3,6,13,16-tetraazaoctadeca-8,10-diyne-1,18-dioate (**8b,** mixture of diastereoisomers).

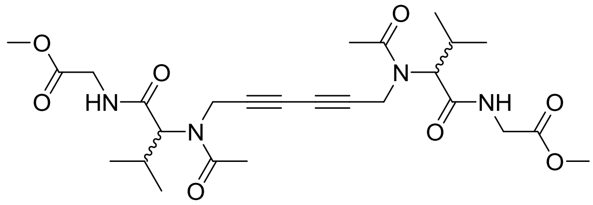

Yield: 80%. Purified by column chromatography. R_f 0.29 (EtOAc / MeOH 19:1). ^{1}H NMR ($CDCl_3$, 400 MHz): δ (ppm) 6.79 (t, J = 5.2 Hz, 4H), 4.65-4.06 (m, 12H), 3.95 (d, J = 5.2 Hz, 8H), 3.73 (s, 12H), 2.28 (m, 16H), 0.99-0.95 (m, 24H). ^{13}C NMR ($CDCl_3$, 100 MHz): δ (ppm) 172.4, 170.6, 169.8, 74.3, 68.2, 62.3,

58.3, 52.3, 41.1, 40.9, 35.1, 31.0, 26.7, 22.1, 19.4, 19.3. HRMS (ESI-pos) m/z calcd for $C_{26}H_{38}N_4NaO_8$ $(M+Na)^+$ 557.2587, found 557.2581.

2,2'-(Hexa-2,4-diyne-1,6-diylbis(acetylazanediyl))bis(*N*-*tert*-butyl-2-(4-methoxyphenyl)acetamide) (**8c**, mixture of diastereoisomers)

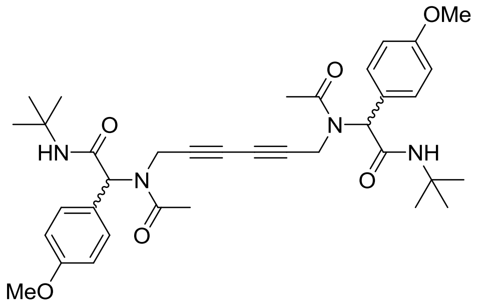

Yield: 99%. Purified by column chromatography. R_f 0.28 (EtOAc). ^{1}H NMR ($CDCl_3$, 400 MHz): δ (ppm) 7.28-7.20 (m, 8H), 6.85-6.81 (m, 8H), 6.08 (s, 4H), 5.83 (bs, 4H), 4.09 (m, 8H), 3.77 (s, 12H), 2.19 (s, 12H), 1.31 (m, 36H). ^{13}C NMR ($CDCl_3$, 100 MHz): δ (ppm) 171.4, 169.5, 169.3, 169.1, 159.7, 159.3, 130.7, 130.6, 128.4, 126.7, 114.2, 74.2, 67.1, 67.0, 59.6, 56.3, 55.3, 55.2, 51.7, 51.6, 42.6, 36.3, 28.6, 28.5, 23.2, 22.1. HRMS (ESI-pos) m/z calcd for $C_{36}H_{46}N_4NaO_6$ $(M+Na)^+$ 653.3315, found 653.3309.

N,N'-(Hexa-2,4-diyne-1,6-diyl)bis(*N*-(1-(*tert*-butylamino)-3-methyl-1-oxobutan-2-yl)benzamide) (**8d**, mixture of diastereoisomers).

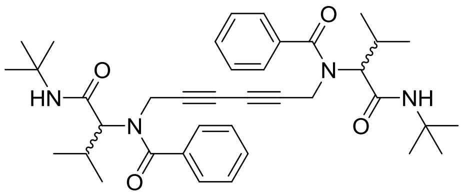

Yield: 91%. Purified by column chromatography. R_f 0.34 (EtOAc / hexane 3:7). ^{1}H NMR ($CDCl_3$, 400 MHz): δ (ppm) 7.42-753 (m, 20H), 6.47 (bs, 4H), 4.45-4.32 (m, 8H), 4.01 (m, 4H), 2.51 (m, 4H), 1.35 (s, 36H), 1.08-1.04 (m, 24H). ^{13}C NMR

(CDCl$_3$, 100 MHz): δ (ppm) 173.4, 169.1, 135.4, 130.5, 128.6, 126.9, 74.9, 68.4, 65.7, 51.3, 37.8, 28.6, 26.8, 19.7, 19.5. HRMS (ESI-pos) m/z calcd for $C_{38}H_{50}N_4NaO_4$ $(M+Na)^+$ 649.3730, found 649.3724.

N,N'-(Hexa-2,4-diyne-1,6-diyl)bis(*N*-(2-(*tert*-butylamino)-2-oxo-1-*p*-tolylethyl)-butyramide) (**8e**, mixture of diastereoisomers).

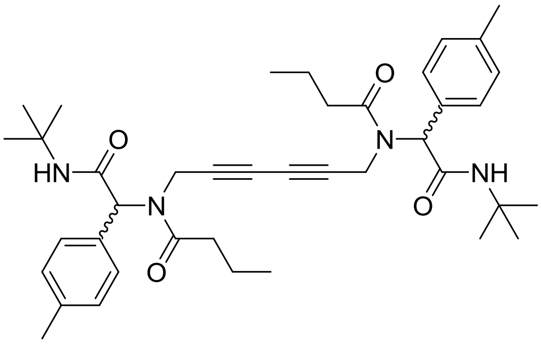

Yield: 99%. Purified by column chromatography. R_f 0.52 (EtOAc / hexane 1:1). ^{1}H NMR (CDCl$_3$, 400 MHz): δ (ppm) 7.28-7.16 (m, 16H), 6.09 (s, 4H), 5.79 (s, 4H), 4.12 (s, 8H), 2.43 (t, J = 6.4 Hz, 8H), 2.34 (s, 12H), 1.71 (m, 8H), 1.34 (s, 36H), 0.98 (t, J = 6.4 Hz, 12H). ^{13}C NMR (CDCl$_3$, 100 MHz): δ (ppm) 173.9, 172.2, 169.1, 138.3, 135.8, 131.8, 129.5, 129.4, 129.3, 127.1, 74.4, 67.1, 60.3, 51.7, 35.7, 35.5, 28.6, 28.5, 21.2, 21.1, 19.0, 18.5, 13.9.. HRMS (ESI-pos) m/z calcd for $C_{40}H_{54}N_4NaO_4$ $(M+Na)^+$ 677.4043, found 677.4037.

N,N'-(Hexa-2,4-diyne-1,6-diyl)bis(*N*-(2-(*tert*-butylamino)-2-oxoethyl)butyramide) (**8f**)

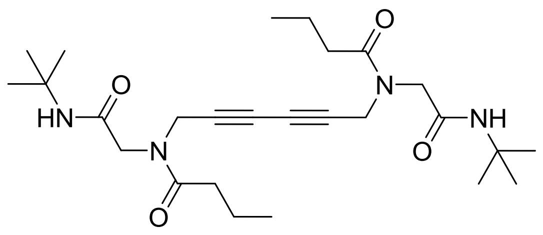

Yield: 99%. Purified by column chromatography. R_f 0.38 (EtOAc / hexane 4:1). ^{1}H NMR ($CDCl_3$, 400 MHz): δ (ppm) 6.82 and 6.10 (s, 2H), 4.28 (m, 4H), 3.96 (m, 4H), 2.41 and 1.66 (m, 4H), 2.31 and 2.25 (m, 4H), 1.37-1.32 (m, 18H), 0.97 (m, 6H). ^{13}C NMR ($CDCl_3$, 100 MHz): δ (ppm) 173.5, 173.4, 167.8, 166.8, 166.6, 74.5, 73.7, 73.5, 72.9, 68.8, 68.5, 68.2, 67.8, 51.9, 51.7, 51.6, 51.3, 51.3, 50.6, 50.5, 43.7, 39.4, 38.2, 36.9, 36.1, 34.8, 28.6, 18.4, 18.3, 13.9, 13.8, 13.7. HRMS (ESI-pos) m/z calcd for $C_{26}H_{42}N_4NaO_4$ $(M+Na)^+$ 497.3104, found 497.3098.

2,2'-(4,13-Dioxo-2,15-dioxa-5,12-diazahexadeca-7,9-diyne-5,12-diyl)bis(*N-tert*-butyl-2-(4-fluorophenyl)acetamide) (**8g**, mixture of diastereoisomers)

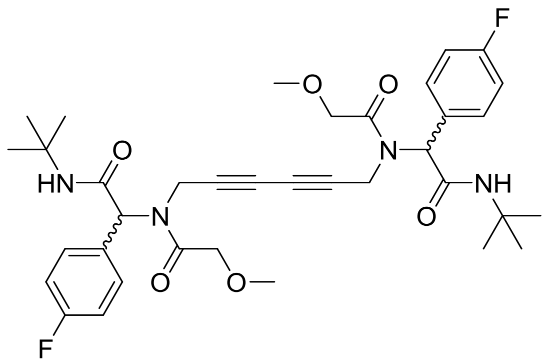

Yield: 97%. Purified by column chromatography. R_f 0.42 (EtOAc). ^{1}H NMR ($CDCl_3$, 400 MHz): δ (ppm) 7.32 (m, 8H), 7.06 (m, 8H), 6.11 (s, 4H), 5.83 (s, 4H), 4.22-4.18 (m, 16H), 3.44 (s, 12H), 1.34 (m, 36H). ^{13}C NMR ($CDCl_3$, 100

MHz): δ (ppm) 170.0, 168.2, 164.0, 161.5, 131.3, 130.2, 115.9, 115.7, 71.2, 59.3, 51.8, 42.6, 34.6, 28.5. HRMS (ESI-pos) m/z calcd for $C_{36}H_{44}F_2N_4NaO_6$ $(M+Na)^+$ 689.3127, found 689.3121.

Di-*tert*-butyl(2R,2'S)-1,1'-(2,2,17,17-tetramethyl-4,15-dioxo-3,6,13,16-tetraaza-octadeca-8,10-diyne-6,13-diyl)bis(1-oxo-3-phenylpropane-2,1-diyl) dicarbamate (**8h**)

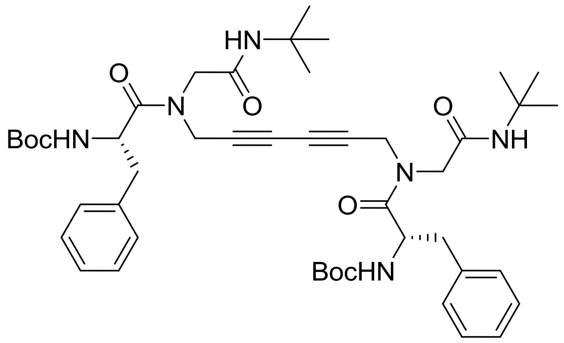

Yield: 99%. Purified by column chromatography. R_f 0.34 (EtOAc). ^{1}H NMR ($CDCl_3$, 400 MHz): δ (ppm) 7.32-7.15 (m, 10H), 6.63 and 6.05 (s, 2H), 5.21 and 5.38 (d, J = 7.6 Hz, 2H), 4.73 (m, 1H), 4.47 (m, 4H), 4.07 (m, 2H), 3.67 (m, 2H), 3.41 (m, 1H), 2.98 (m, 4H), 1.38-1.32 (m, 36H). ^{13}C NMR ($CDCl_3$, 100 MHz): δ (ppm) 172.4, 172.2, 167.0, 166.9, 166.4, 166.3, 155.5, 155.3, 135.8, 135.5, 129.4, 128.8, 128.7, 127.4, 127.2, 80.2, 80.0, 74.1, 72.9, 720, 69.7, 68.5, 68.0, 52.0, 51.8, 51.4, 50.8, 50.6, 39.0, 38.7, 38.5, 36.4, 28.7, 28.6, 28.2. HRMS (ESI-pos) m/z calcd for $C_{46}H_{64}N_6NaO_8$ $(M+Na)^+$ 851.4683, found 851.4634.

2,2'-(2,5,14,17-Tetraoxo-1,18-diphenyl-3,6,13,16-tetraazaoctadeca-8,10-diyne-6,13-diyl)bis(*N*-tert-butyl-3-methylbutanamide) (**8i**, mixture of diastereoisomers)

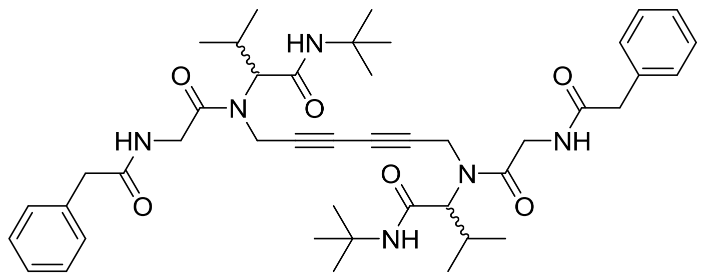

Yield: 96%. Purified by column chromatography. R_f 0.41 (EtOAc / hexane 4:1). ^{1}H NMR ($CDCl_3$, 400 MHz): δ (ppm) 8.68 and 8.42 (s, 4H), 7.39-7.20 (m, 20H), 6.45 and 5.66 (s, 4H), 4.72-4.01 (m, 14H), 3.75 (m, 2H), 3.59 (m, 8H), 2.98 (s, 4H), 2.18 (m, 4H), 1.25-1.30 (m, 36H), 0.96-0.80 (m, 24H). ^{13}C NMR ($CDCl_3$, 100 MHz): δ (ppm) 171.9, 171.7, 170.1, 169.9, 169.6, 168.6, 168.5, 168.4, 168.1, 168.0, 135.2, 135.1, 134.5, 134.4, 129.5, 129.3, 128.9, 128.6, 128.5, 127.4, 126.9, 75.7, 75.1, 73.8, 72.1, 69.4, 68.6, 66.5, 66.0, 63.3, 51.7, 51.6, 51.5, 51.4, 43.5, 43.2, 43.1, 42.9, 41.9, 41.8, 41.7, 41.7, 33.2, 33.1, 33.0, 32.7, 28.7, 28.6, 28.5, 27.2, 20.0, 19.3, 19.2, 19.1. HRMS (ESI-pos) m/z calcd for $C_{44}H_{60}N_6NaO_6$ $(M+Na)^+$ 791.4472, found 791.4466.

Dibenzyl (2,2,17,17-tetramethyl-4,15-dioxo-3,6,13,16-tetraazaoctadeca-8,10-diyne-6,13-diyl)bis(2-oxoethane-2,1-diyl)dicarbamate (**8j**)

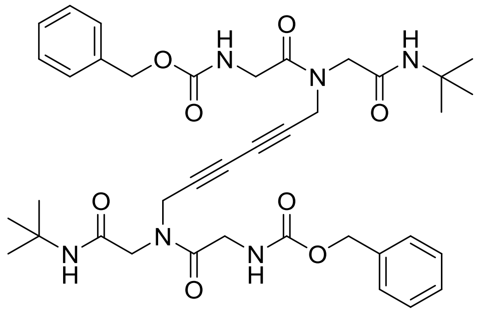

Yield: 99%. Purified by column chromatography. R_f 0.43 (EtOAc). ^{1}H NMR ($CDCl_3$, 400 MHz): δ (ppm) 7.33 (m, 10H), 6.36 and 6.11 (s, 2H), 6.19 and 5.95 (s, 2H), 5.83 (s, 2H), 5.09 (m, 2H), 4.35- 3.89 (m, 12H), 1.34 and 1.31 (m, 18H). ^{13}C NMR ($CDCl_3$, 100 MHz): δ (ppm) 169.3, 169.1, 168.9, 166.9, 165.9, 165.8, 156.3, 136.3, 128.5, 128.2, 127.0, 74.4, 73.5, 72.9, 72.0, 69.5, 69.1, 68.4, 67.0, 52.0, 51.2, 50.1, 49.6, 42.6, 38.4, 36.8, 28.6. HRMS (ESI-pos) m/z calcd for $C_{38}H_{48}N_6NaO_8$ $(M+Na)^+$ 739.3431, found 739.3425.

Combinatorial approach to dimers

In a 10 mL round botton flask, to stirred solution of the alkynes **7f**, **7j** and **7h** (0.25 mmol each), CuCl (0.07 mmol / 5 mol%) was added. The contents were stirred at 90 ºC under air atmosphere. After 24 h the reaction mixture was diluted with ethyl acetate (10 mL) and filtered through a Celite plug. The solvent was removed under reduced pressure. The crude material analyzed by HPLC.

Biological activity assay

The antibacterial activity against *Bacillus subtilis* was determined with a fluorescence based antibacterial growth inhibition assay. The fluorescence was measured on a microtiter plate reader GENios Pro (Fa. Tecan, excitation, 510 nm; emission, 535 nm). The *Bacillus subtilis* strain 168 (P_{AbrB}-IYFP) was maintained on TY (tryptone-yeast extract) medium supplemented with 1 % Bacto-tryptone, 0.5 % Bacto-yeast extract, 1 % NaCl and chloramphenicol (5 µg ml^{-1}). Details of the assay are published:

Katharina Michels, Ramona Heinke, Oscar P. Kuipers, Norbert Arnold and

Ludger A. Wessjohann "A Fluorescence-based Bioassay for Antibacterials and its Application in Screening Natural Product Extracts", *J. Antibiot.* submitted.

Table S1

Compound	Growth inhibition[a] in % at 1 µM[d]	Standard deviation[d]	Growth inhibition in % at 10 µM[d]	Standard deviation[d]
**8a**	26.4	18.9	40.9	24.5
**8b**	44.0	26.7	52.3	27.8
**8c**	1.3	5.1	23.7	12.7
**8d**	44.0	21.8	54.9	19.1
**8e**	29.3	11.4	34.1	16.2
**8f**	2.3	13.5	30.1	21.4
**8g**	36.2	15.5	41.2	17.1
**8h**	43.9	23.0	49.9	23.5
**8i**	39.2	12.6	44.3	10.4
**8j**	23.2	17.0	57.6	26.5
**Std.**[b]	70.8	4.5	NP[c]	NP[c]

[a] Measured after 15 h
[b] Erythromycin
[c] Not performed.
[d] Mean values of two trials involving 3 replicates

Figures of ^{1}H and ^{13}C NMR spectra

Please note that spectra of *N*-alkyl-amides (peptoids) like Ugi products display double signal sets in NMR due to interconvertible isomers with *s-cis* and *s-trans* amide bonds. Depending on substitution pattern, solvent and temperature, the equilibrium between these forms is shifted and may lead to broadened or doubled peaks of varied intensity.

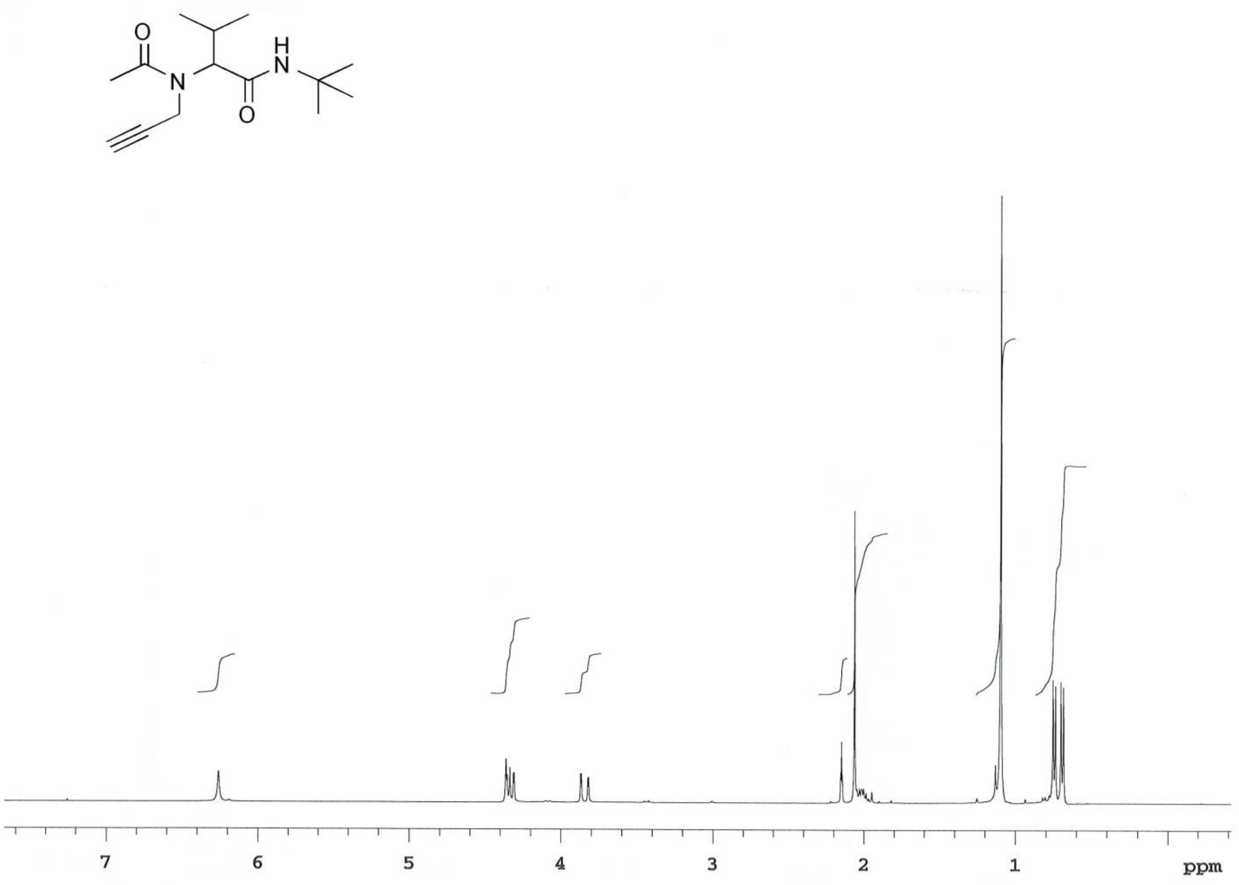

Figure S1: ^{1}H NMR spectrum of compound **7a**

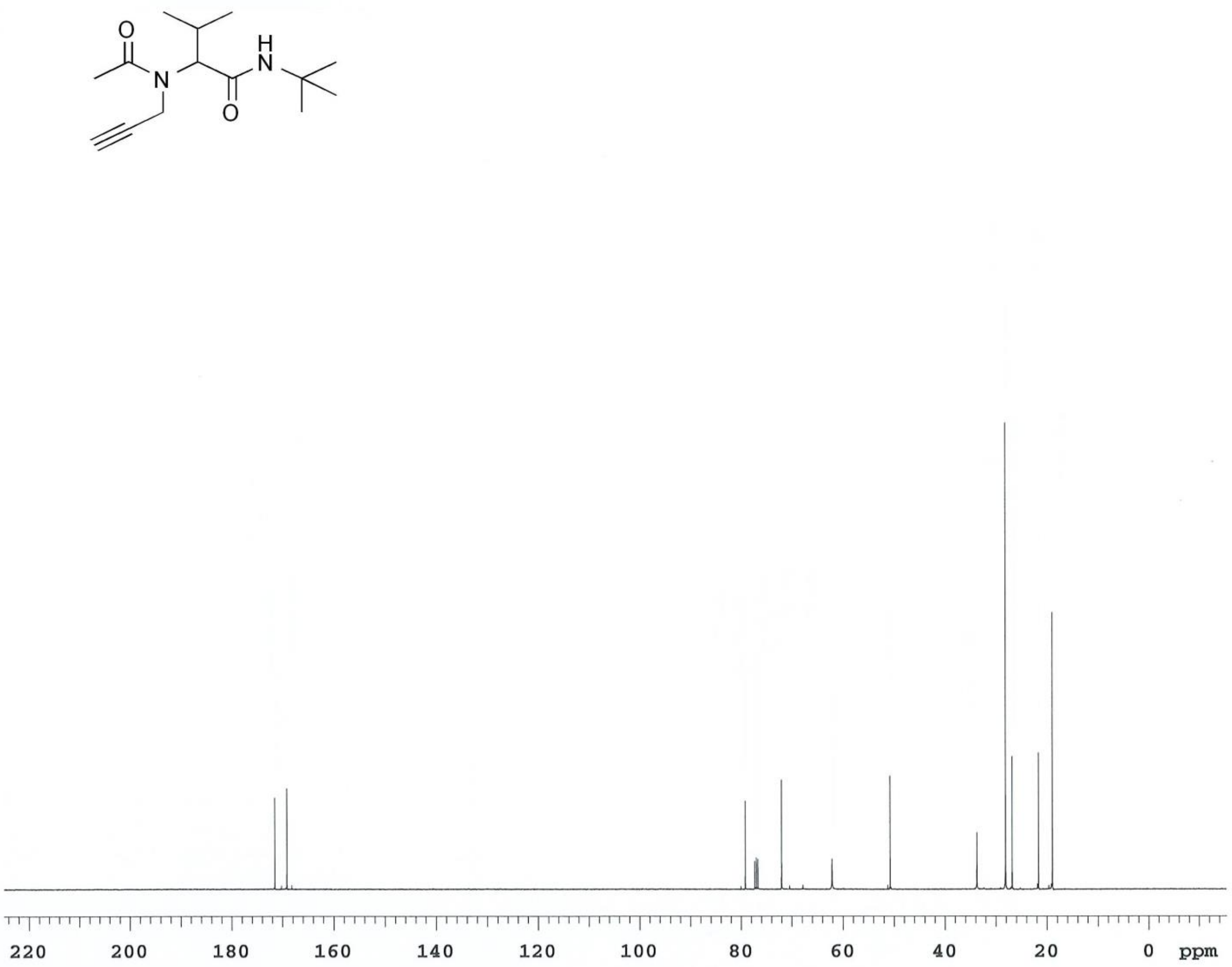

Figure S2: ^{13}C NMR spectrum of compound **7a**

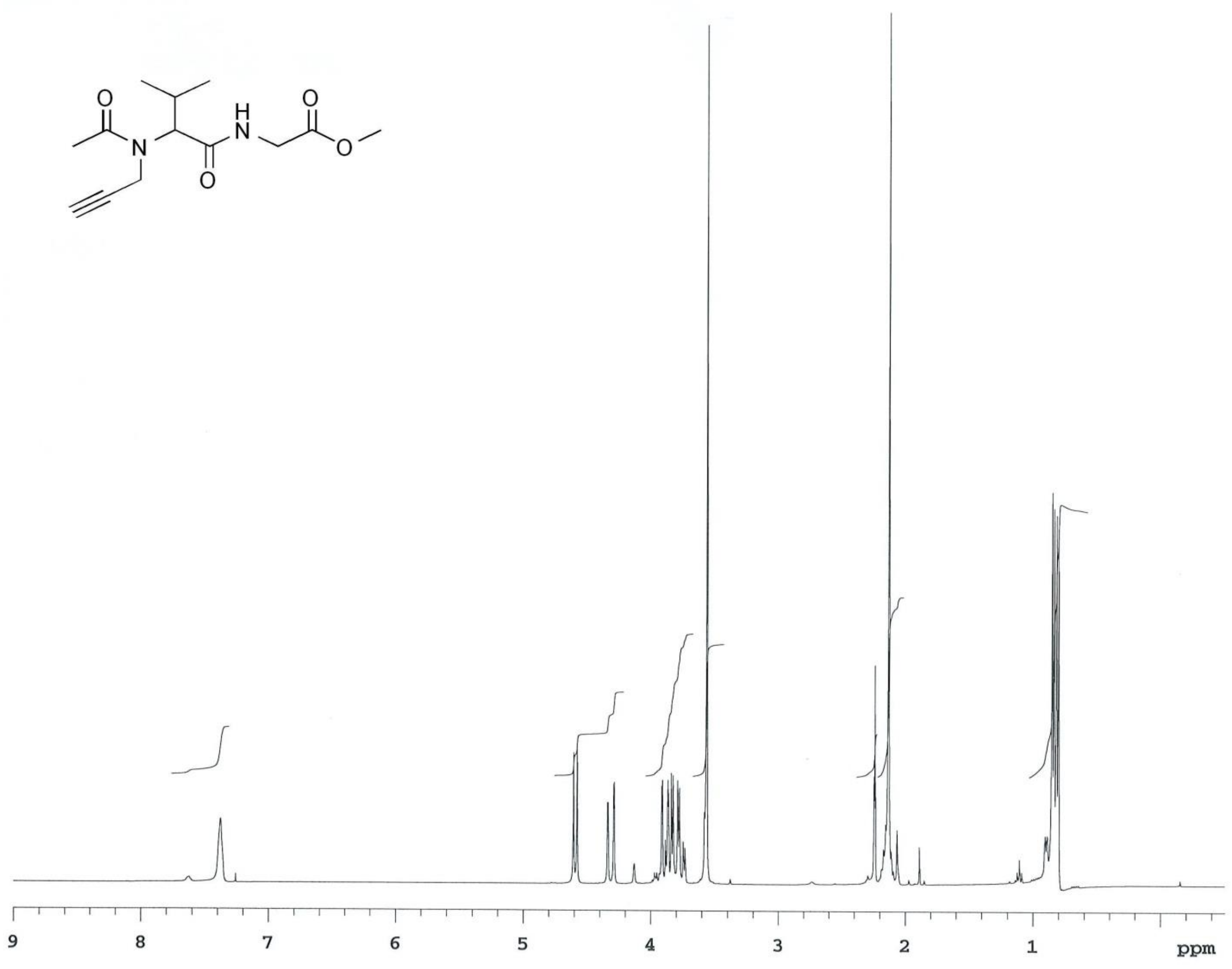

Figure S3: ^{1}H NMR spectrum of compound **7b**

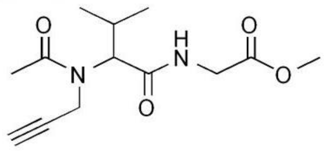

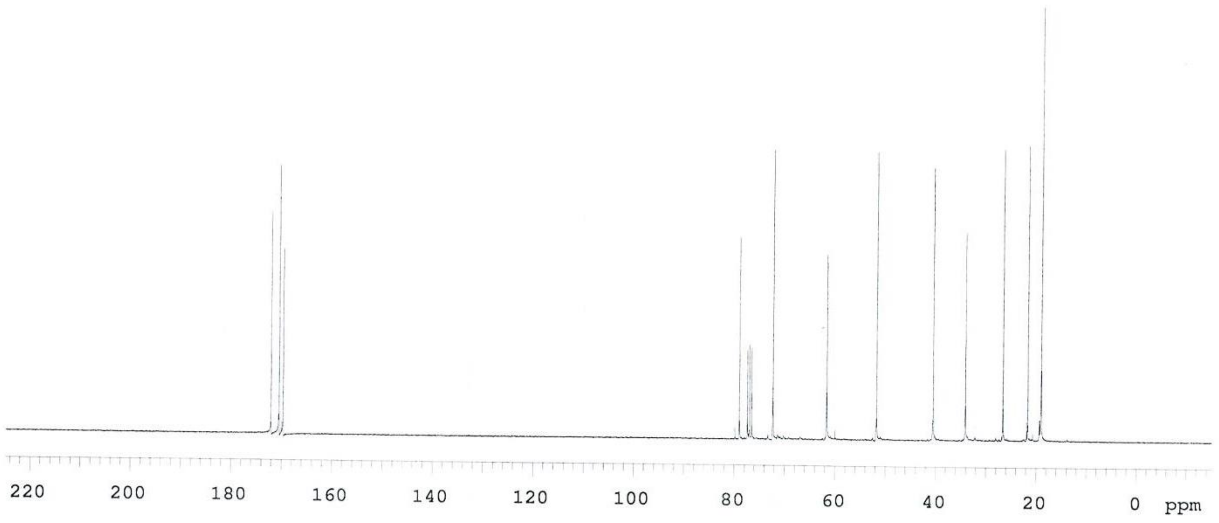

Figure S4: ^{13}C NMR spectrum of compound **7b**

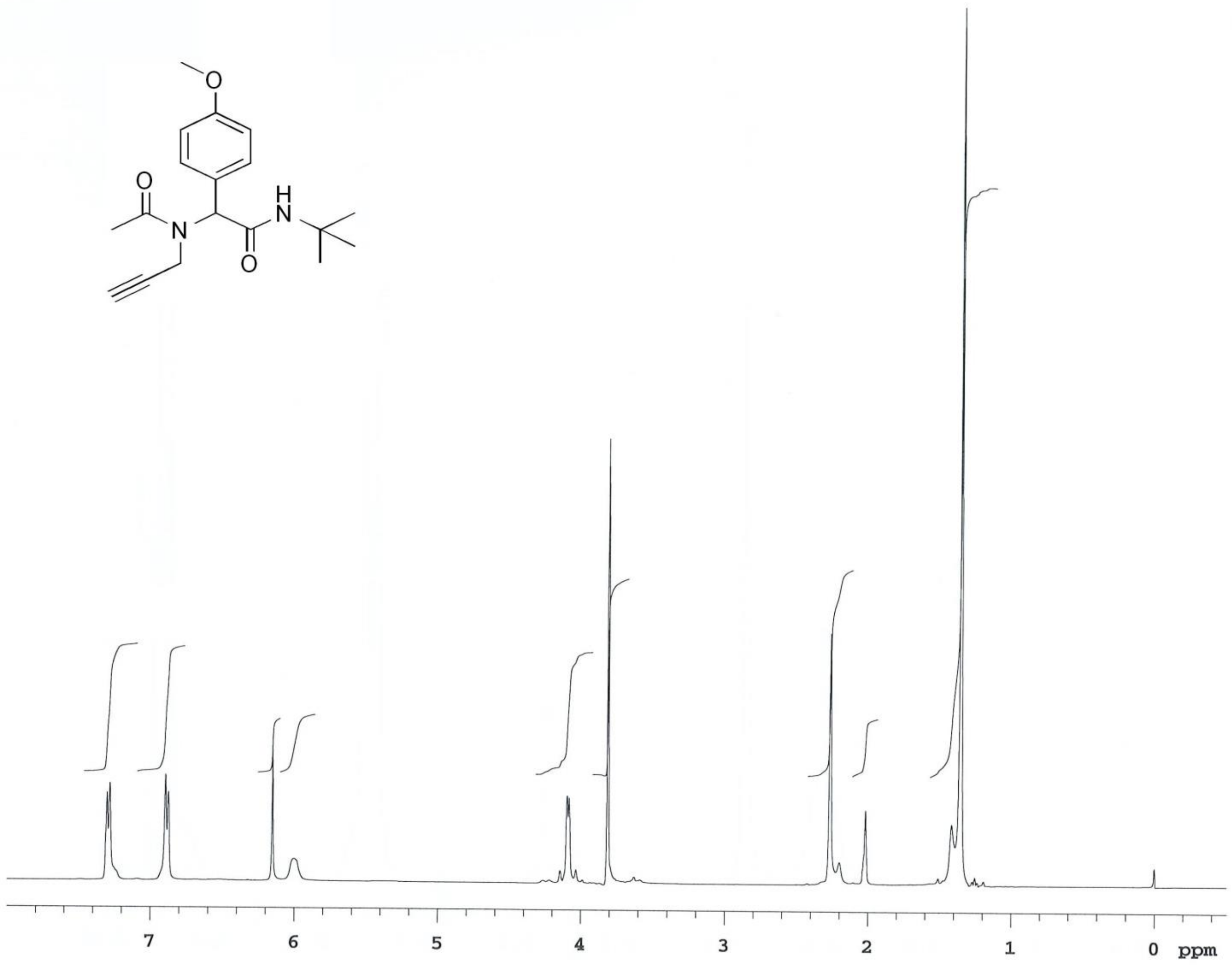

Figure S5: ^{1}H NMR spectrum of compound **7c**

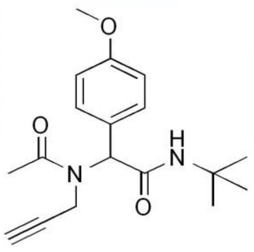

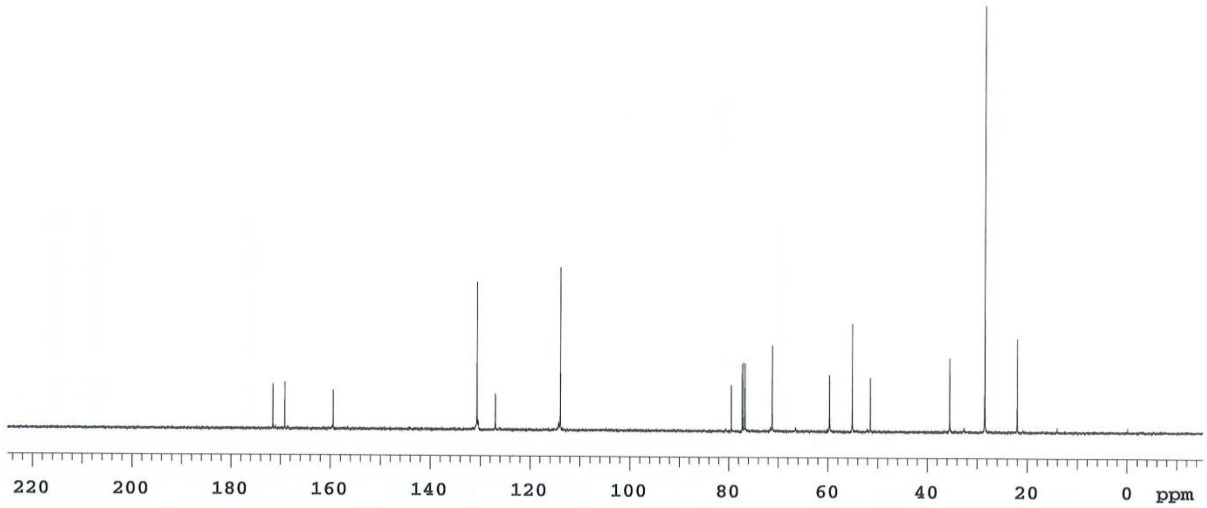

Figure S6: ^{13}C NMR spectrum of compound **7c**

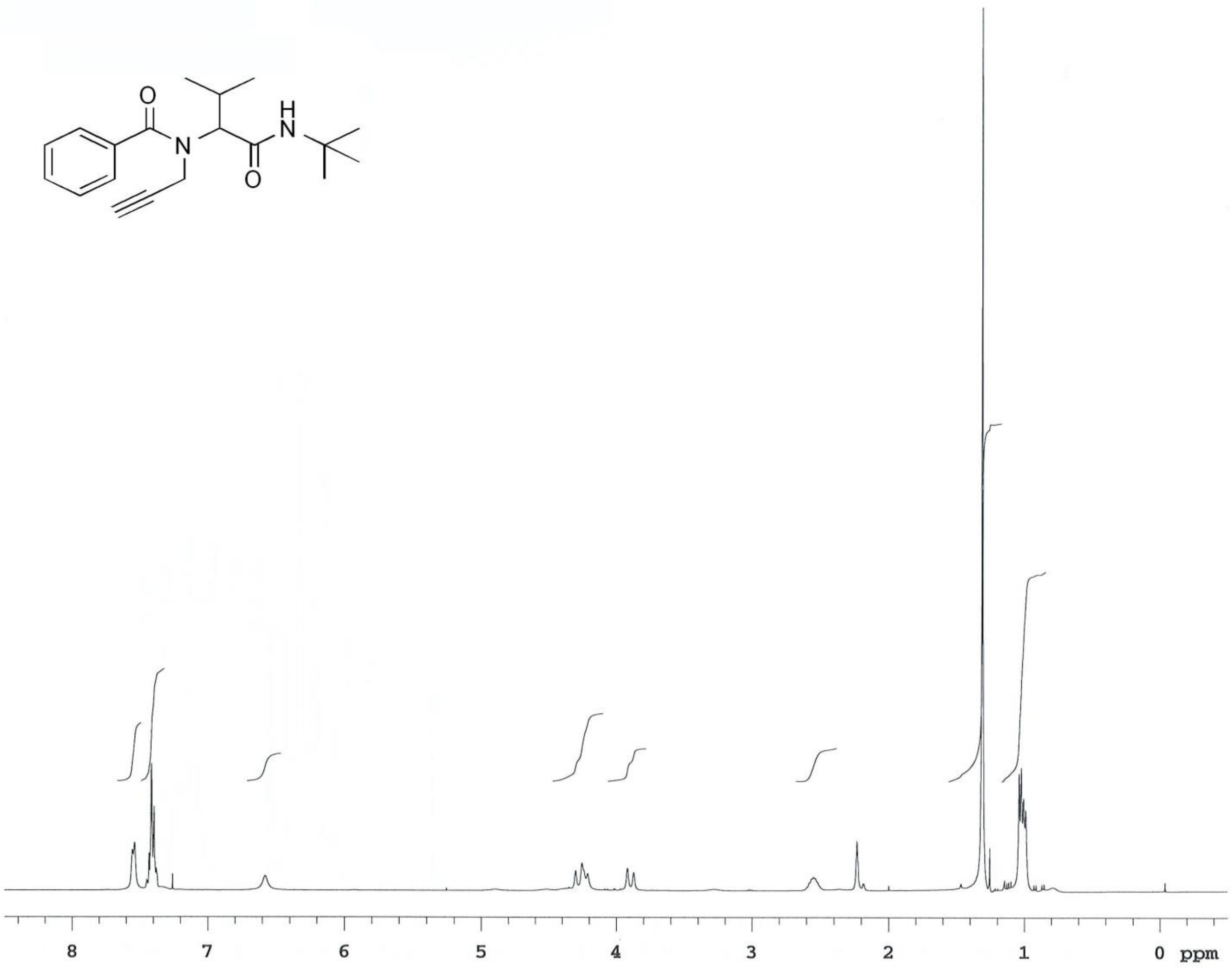

Figure S7: ^{1}H NMR spectrum of compound **7d**

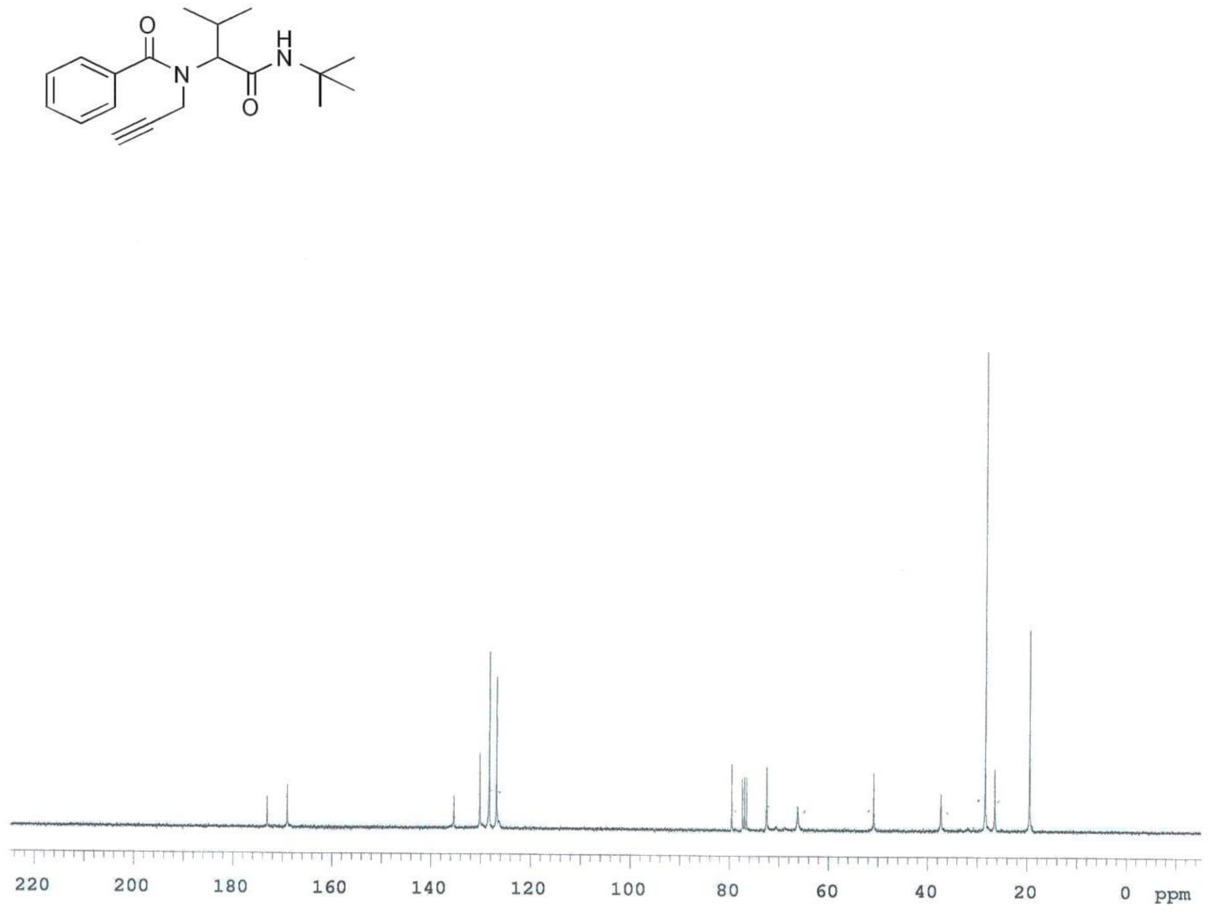

Figure S8: ^{13}C NMR spectrum of compound **7d**

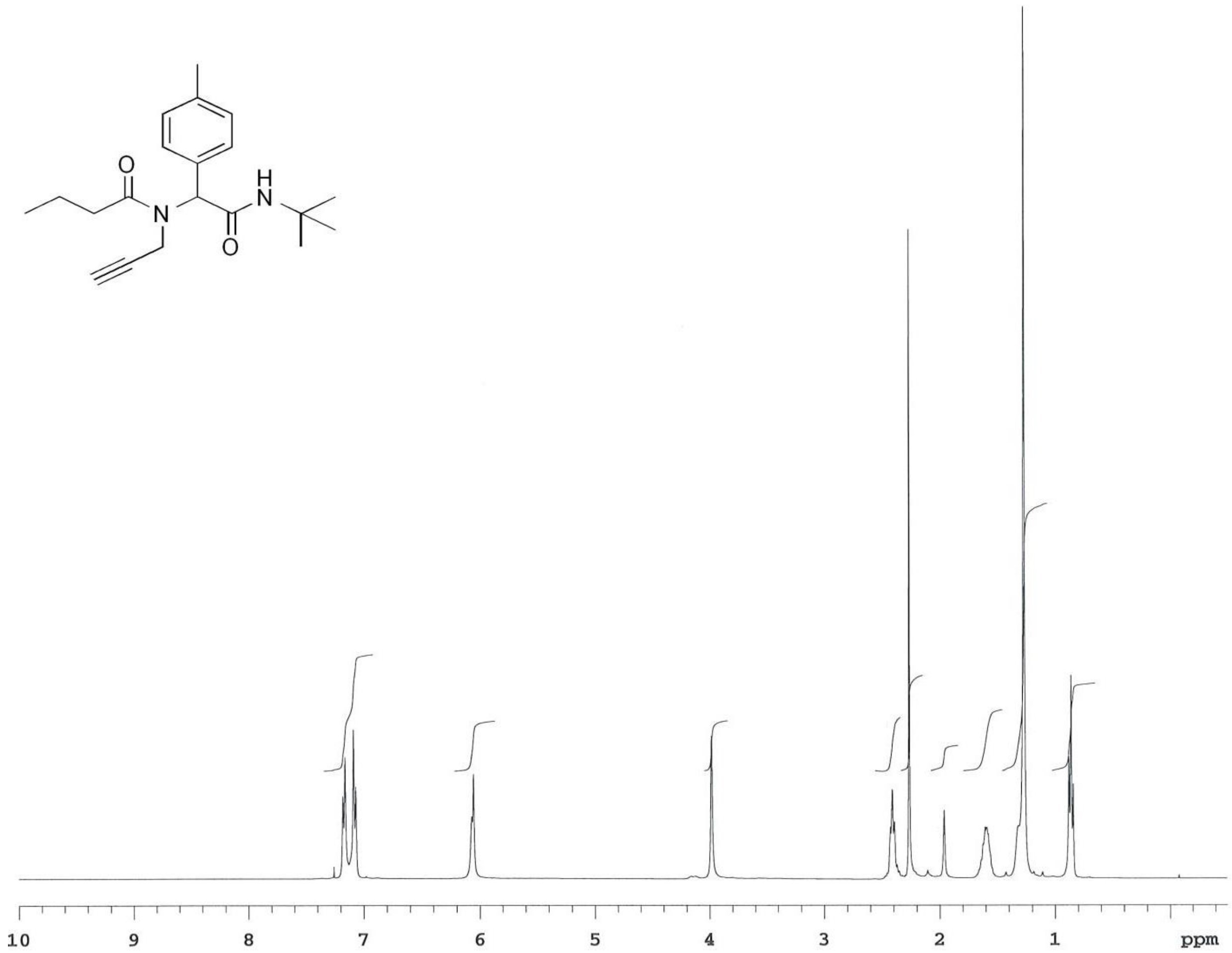

Figure S9: ^{1}H NMR spectrum of compound **7e**

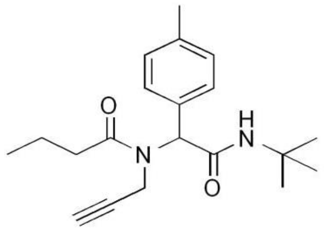

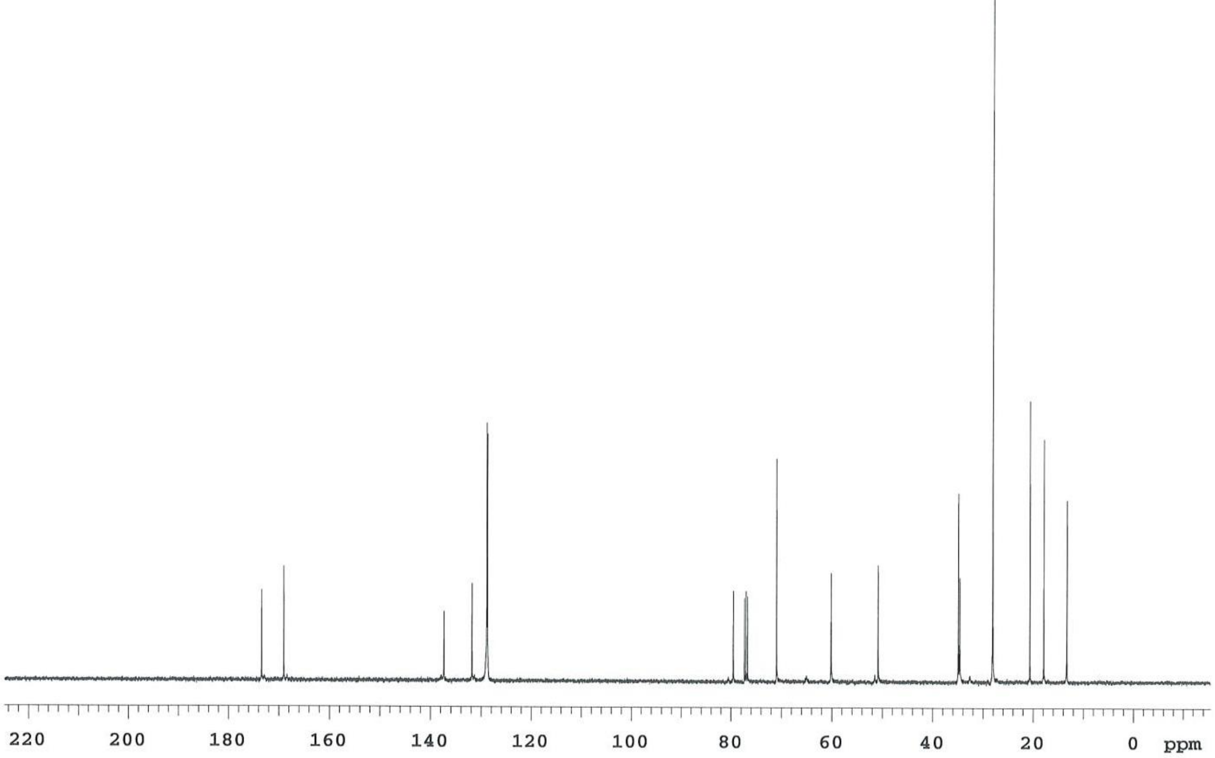

Figure S10: ^{13}C NMR spectrum of compound **7e**

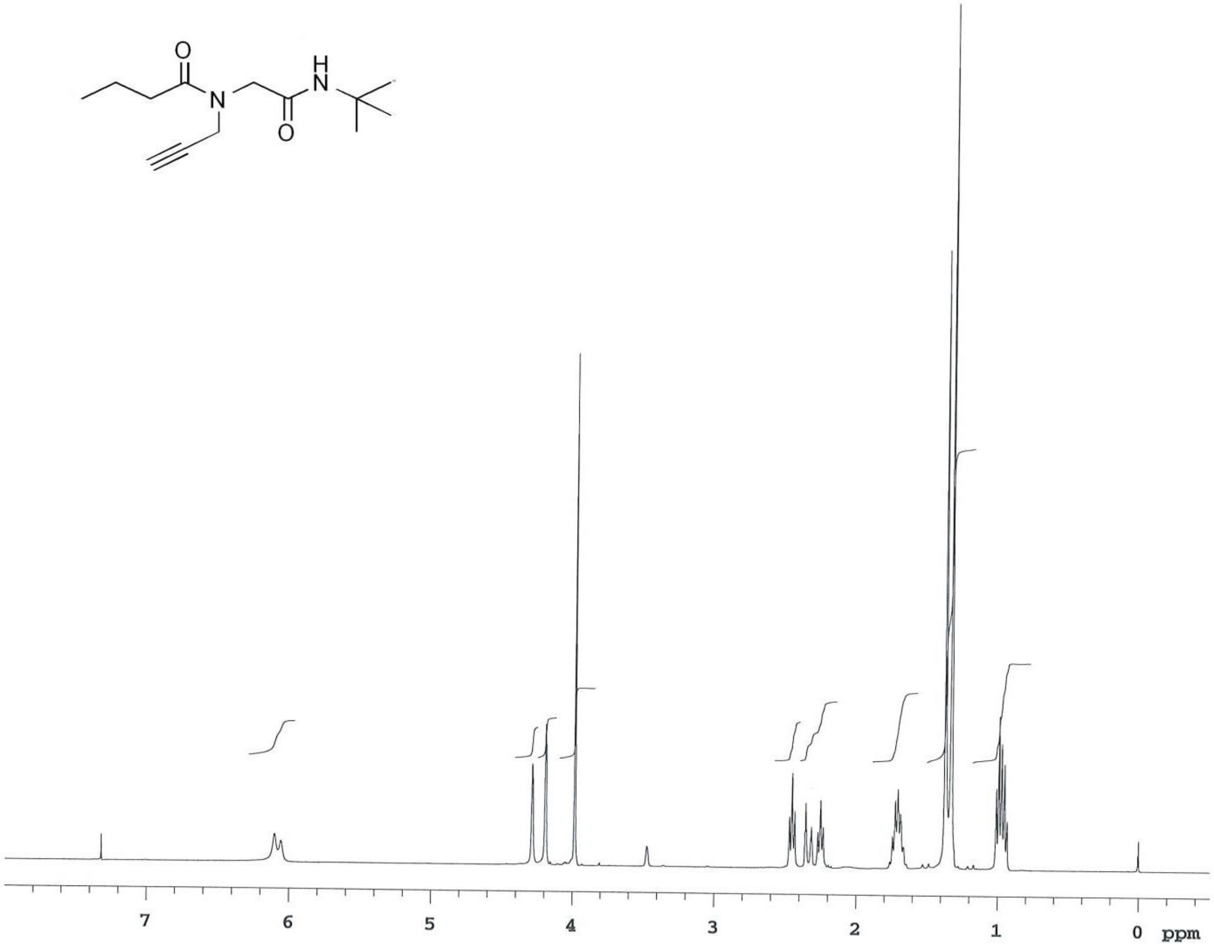

Figure S11: ^{1}H NMR spectrum of compound **7f**

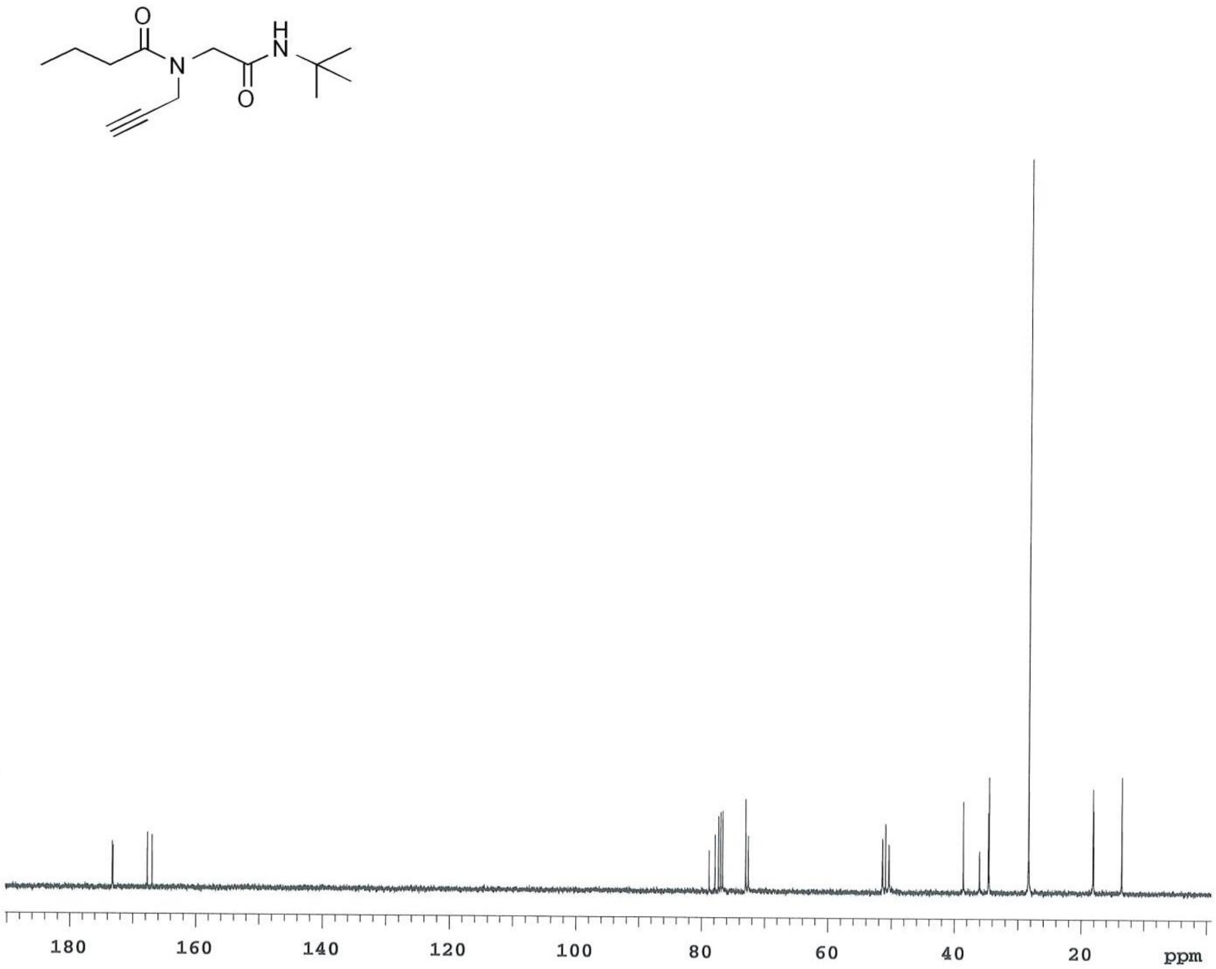

Figure S12: ^{13}C NMR spectrum of compound **7f**

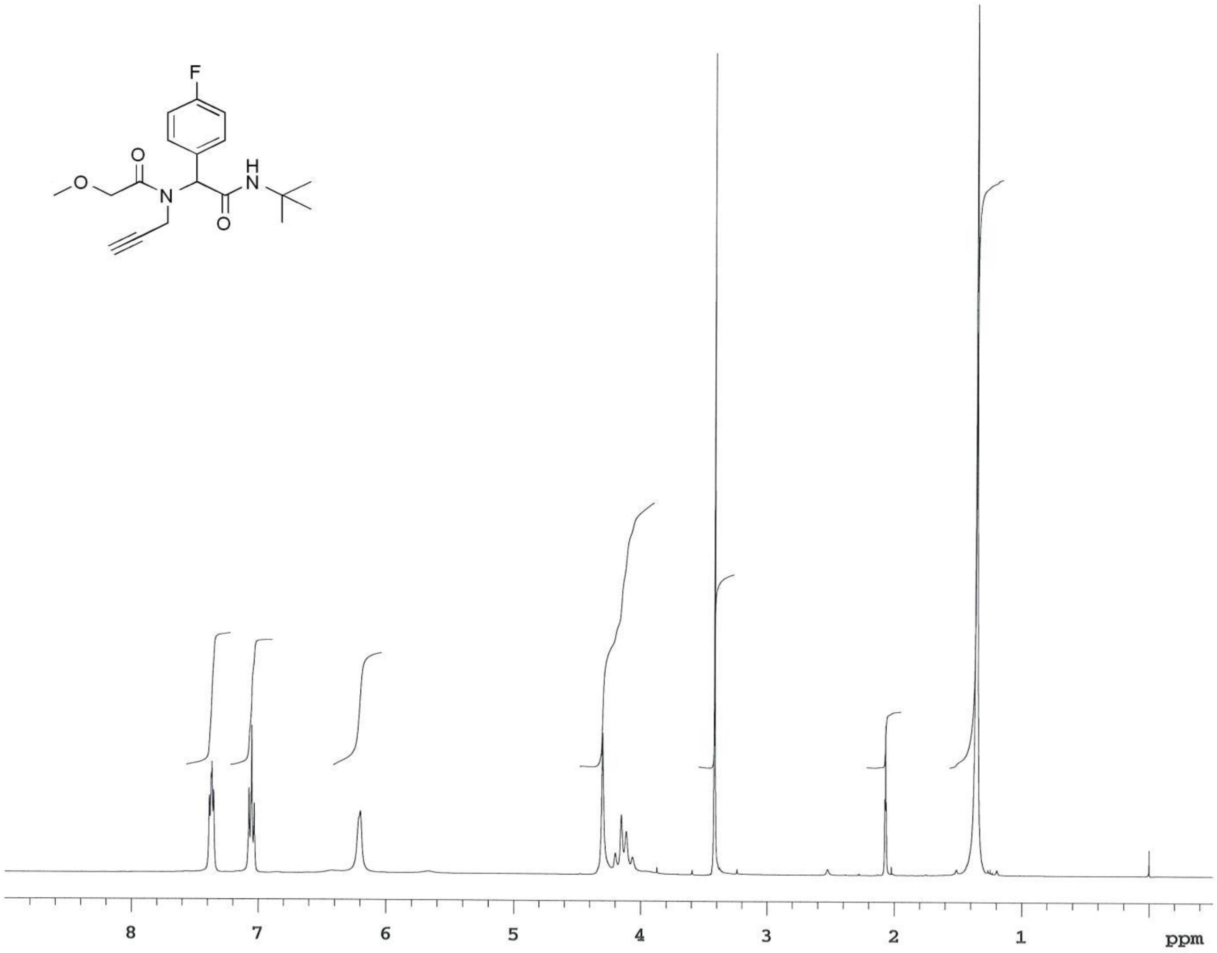

Figure S13: ^{1}H NMR spectrum of compound **7g**

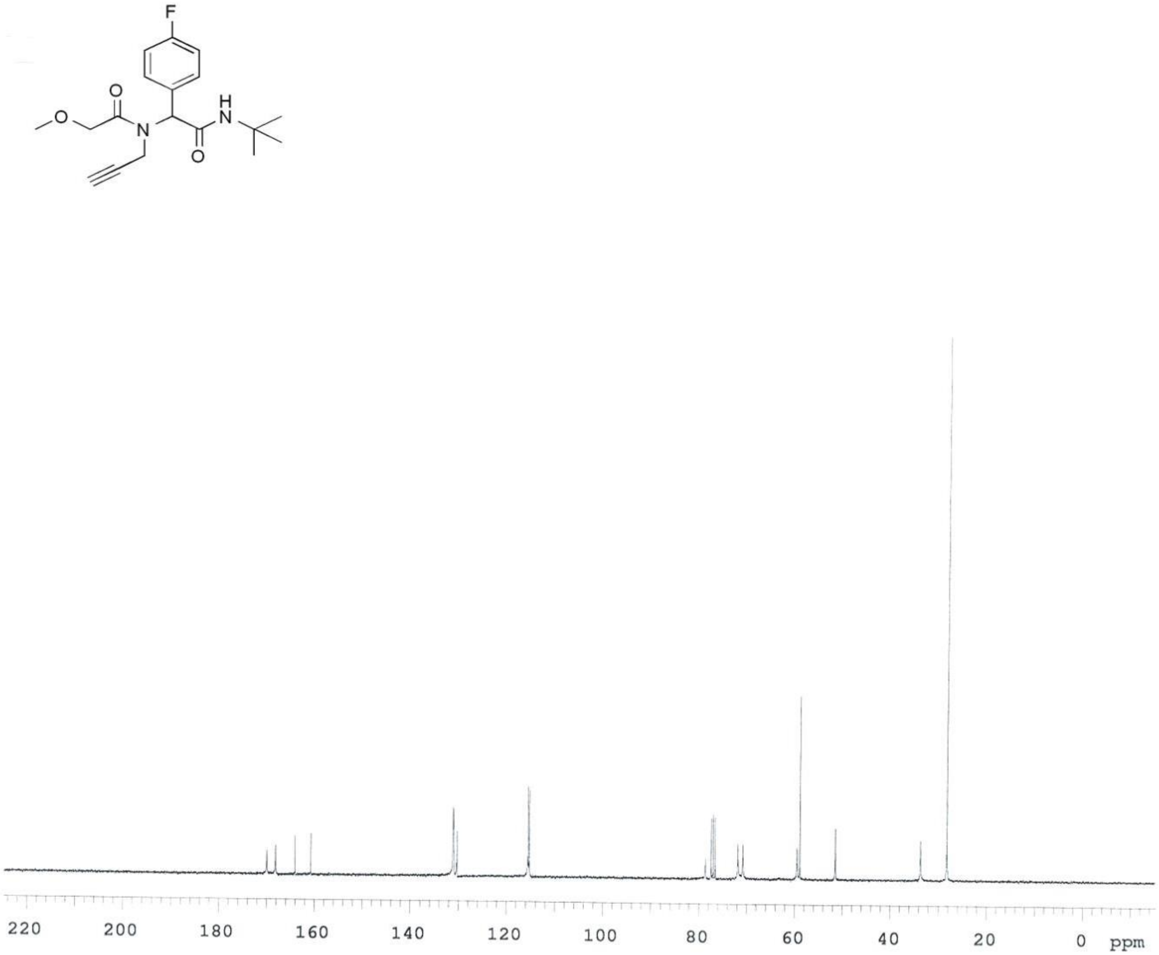

Figure S14: ^{13}C NMR spectrum of compound **7g**

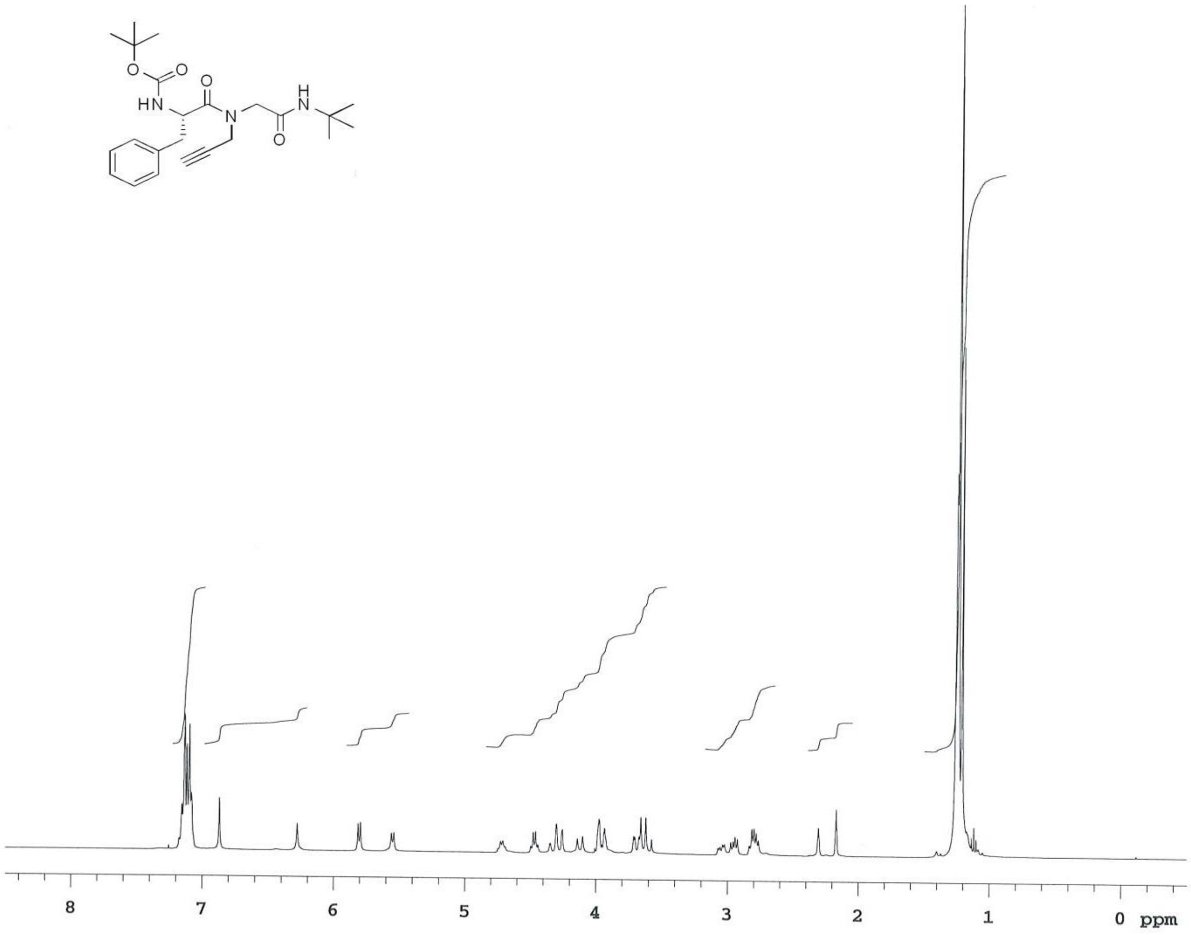

Figure S15: ^{1}H NMR spectrum of compound **7h**

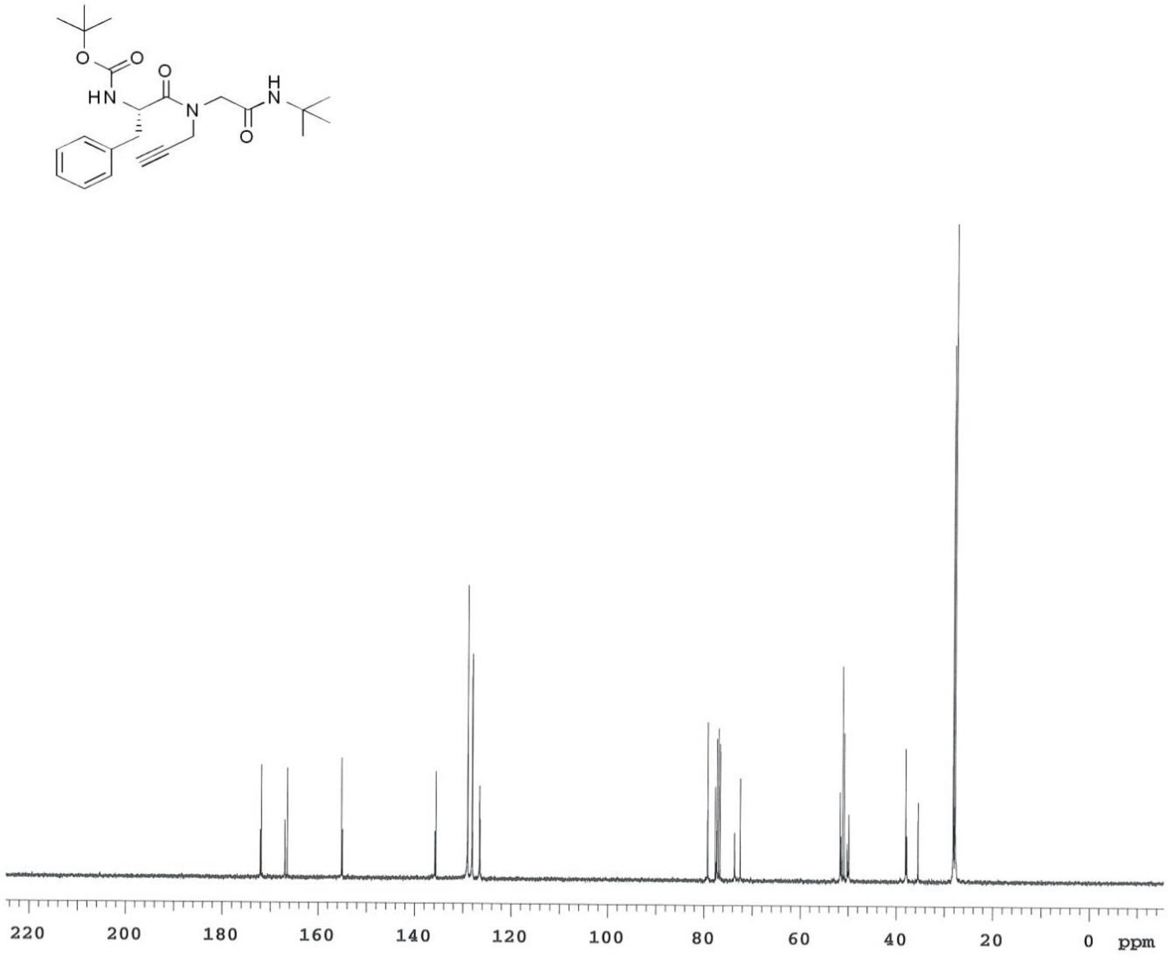

Figure S16: ^{13}C NMR spectrum of compound **7h**

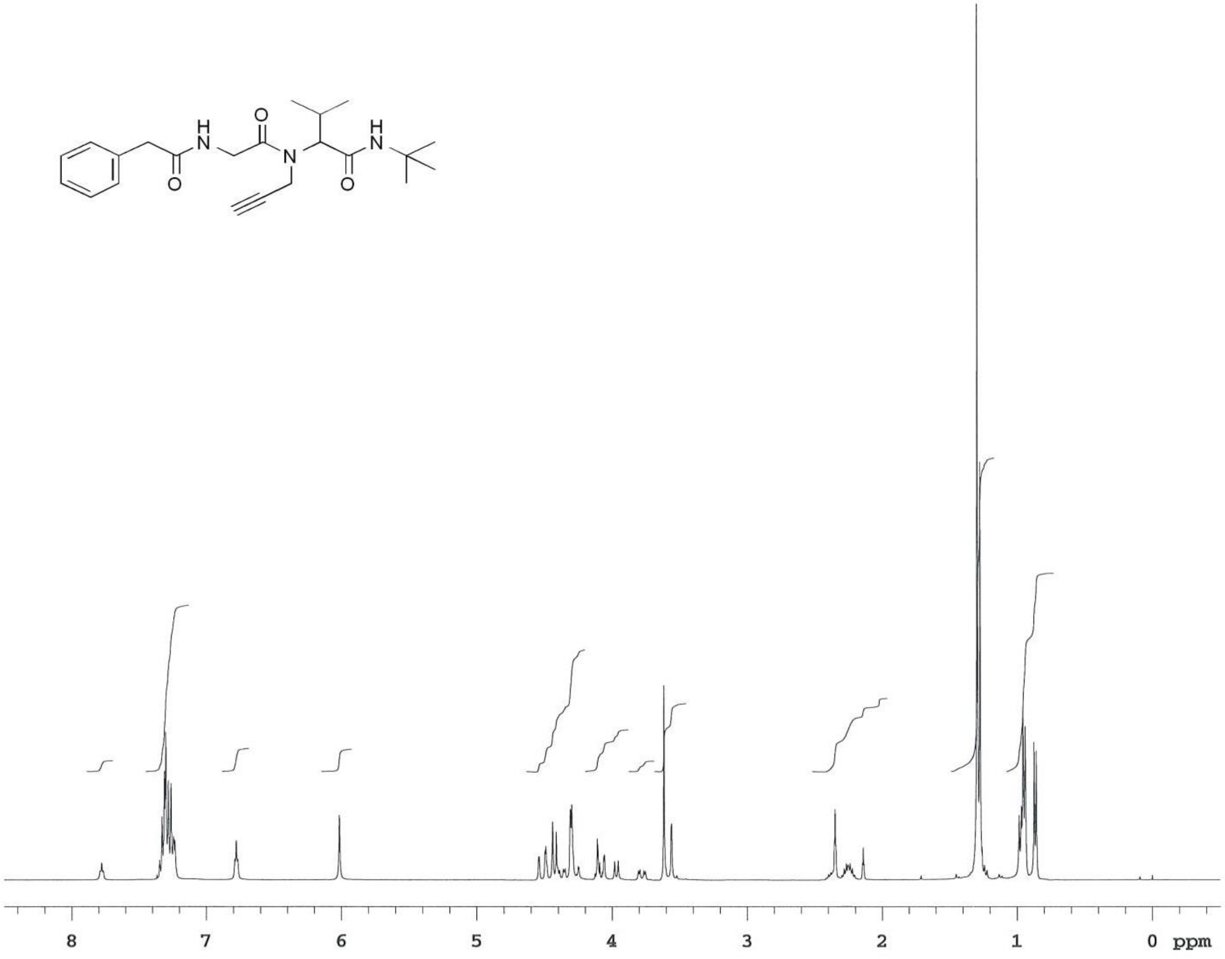

Figure S17: ^{1}H NMR spectrum of compound **7i**

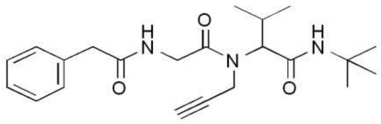

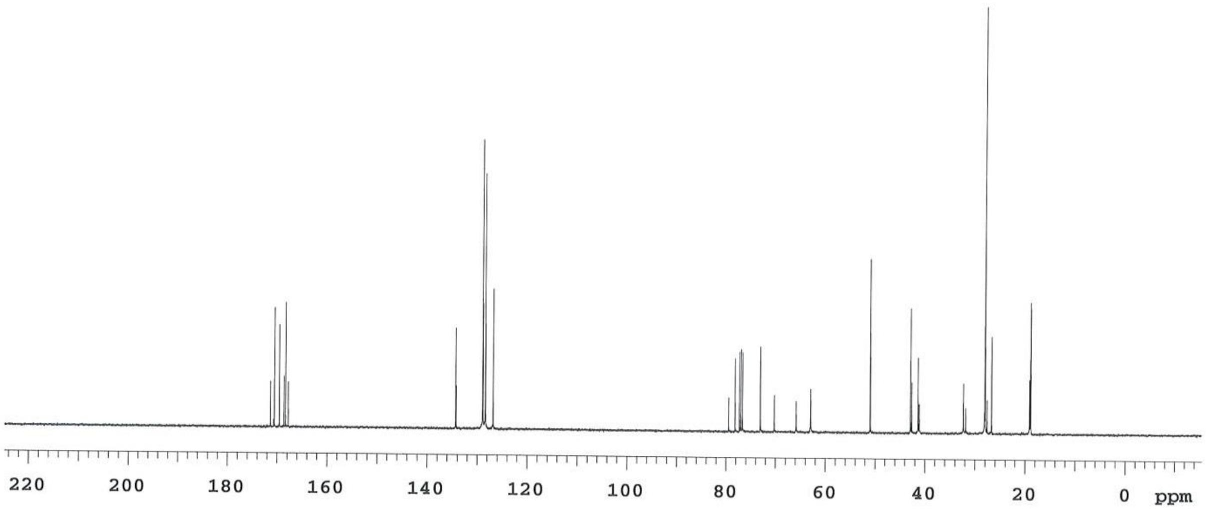

Figure S18: ^{13}C NMR spectrum of compound **7i**

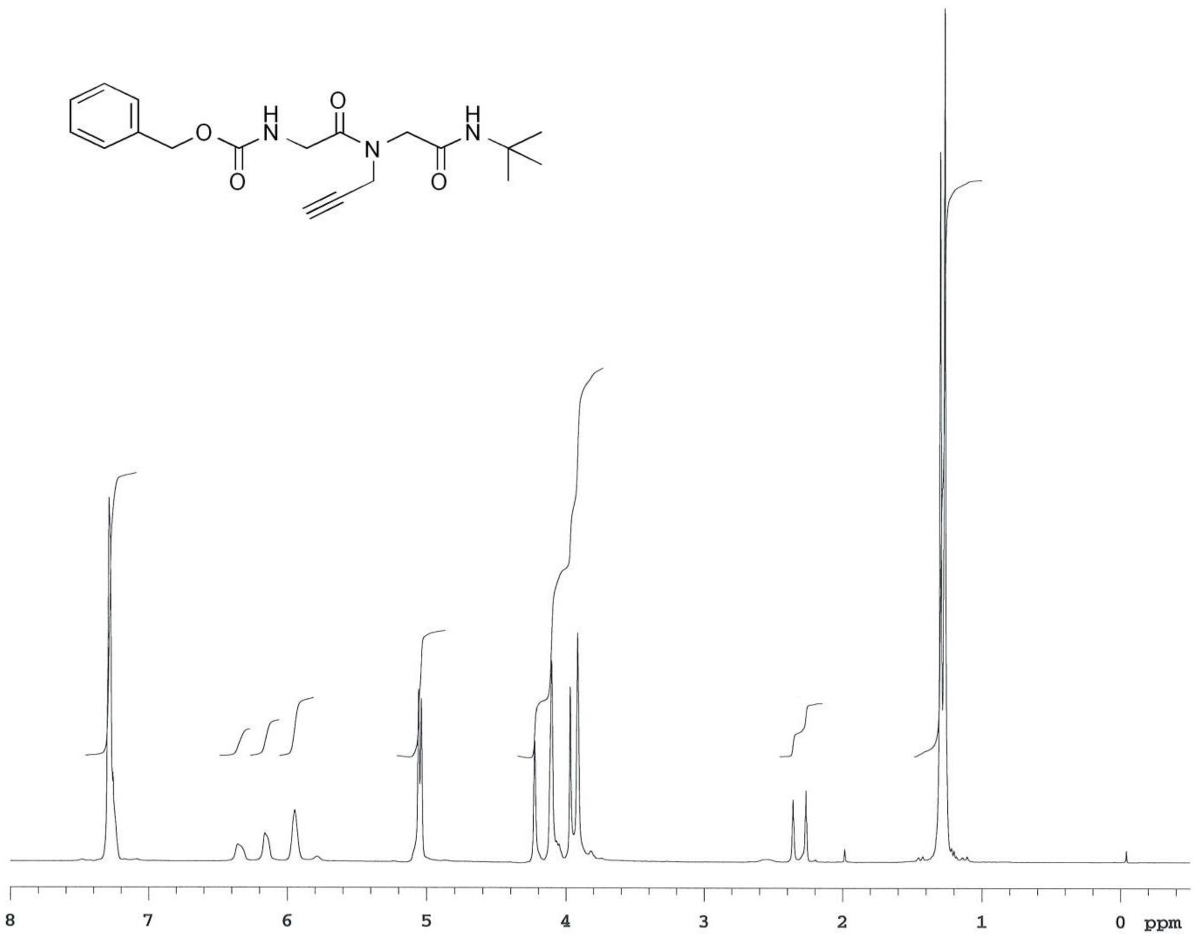

Figure S19: ^{1}H NMR spectrum of compound **7j**

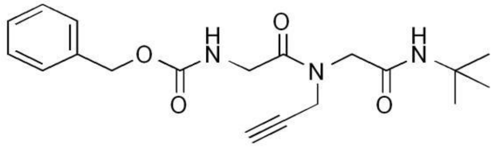

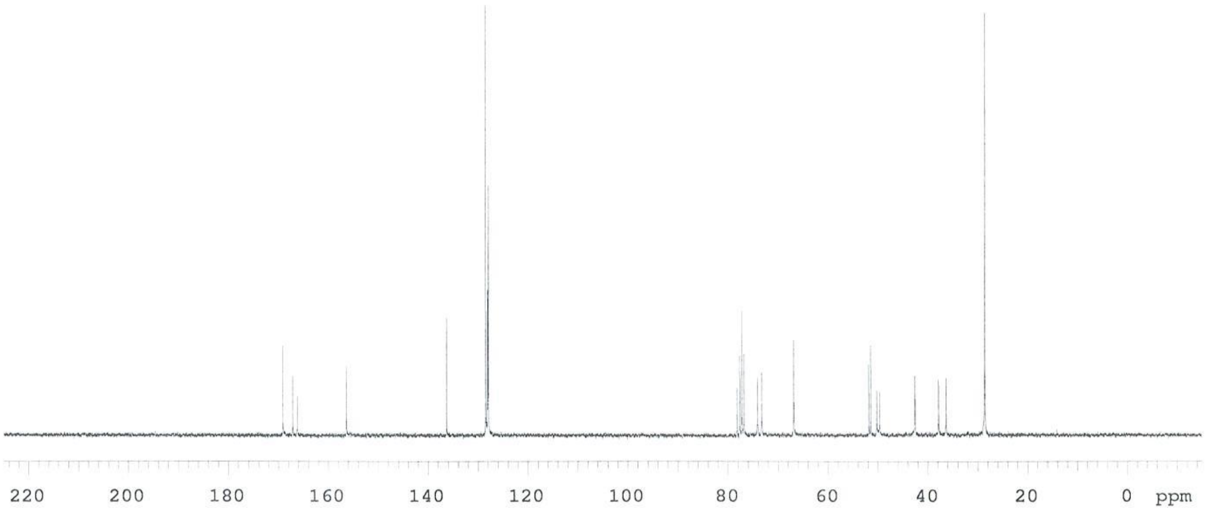

Figure S20: ^{13}C NMR spectrum of compound **7j**

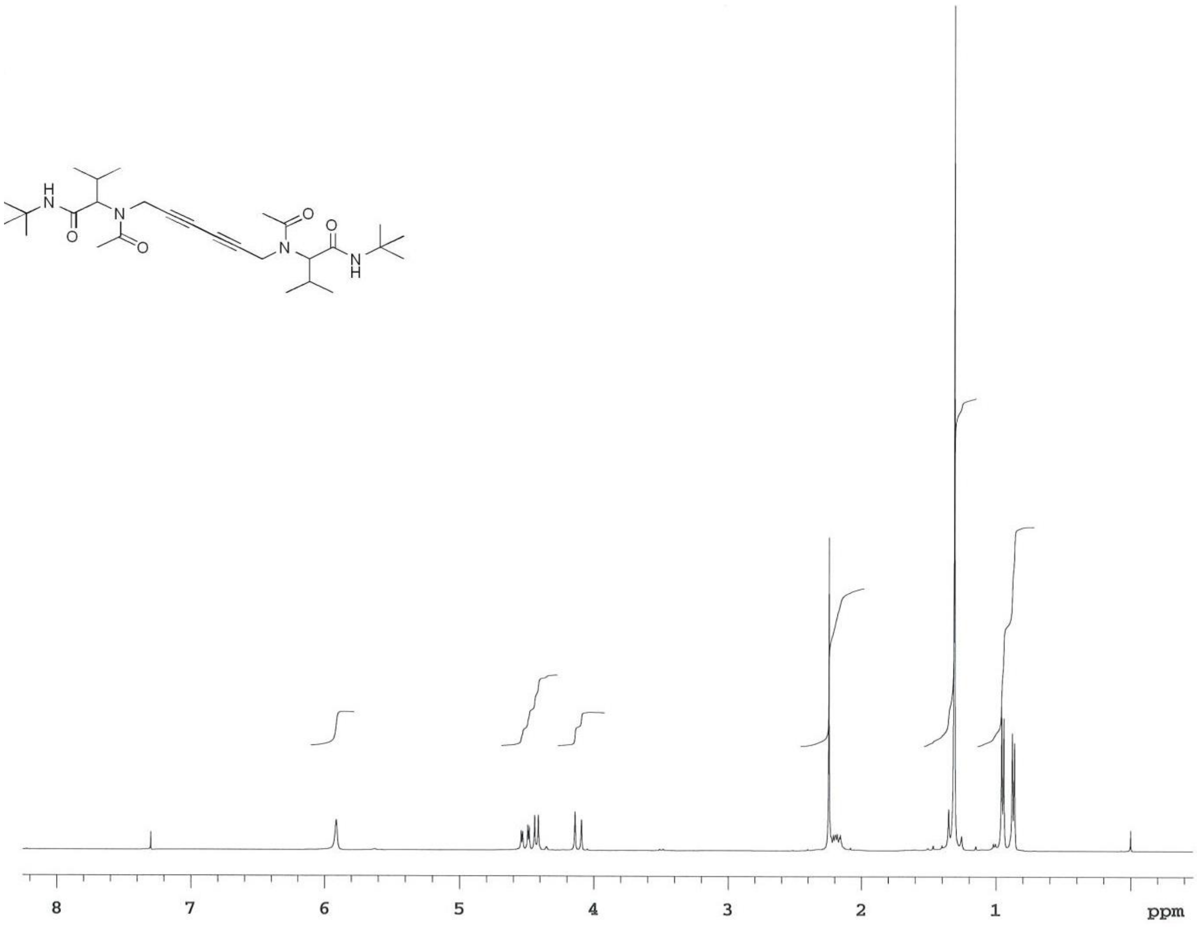

Figure S21: ^{1}H NMR spectrum of compound **8a**

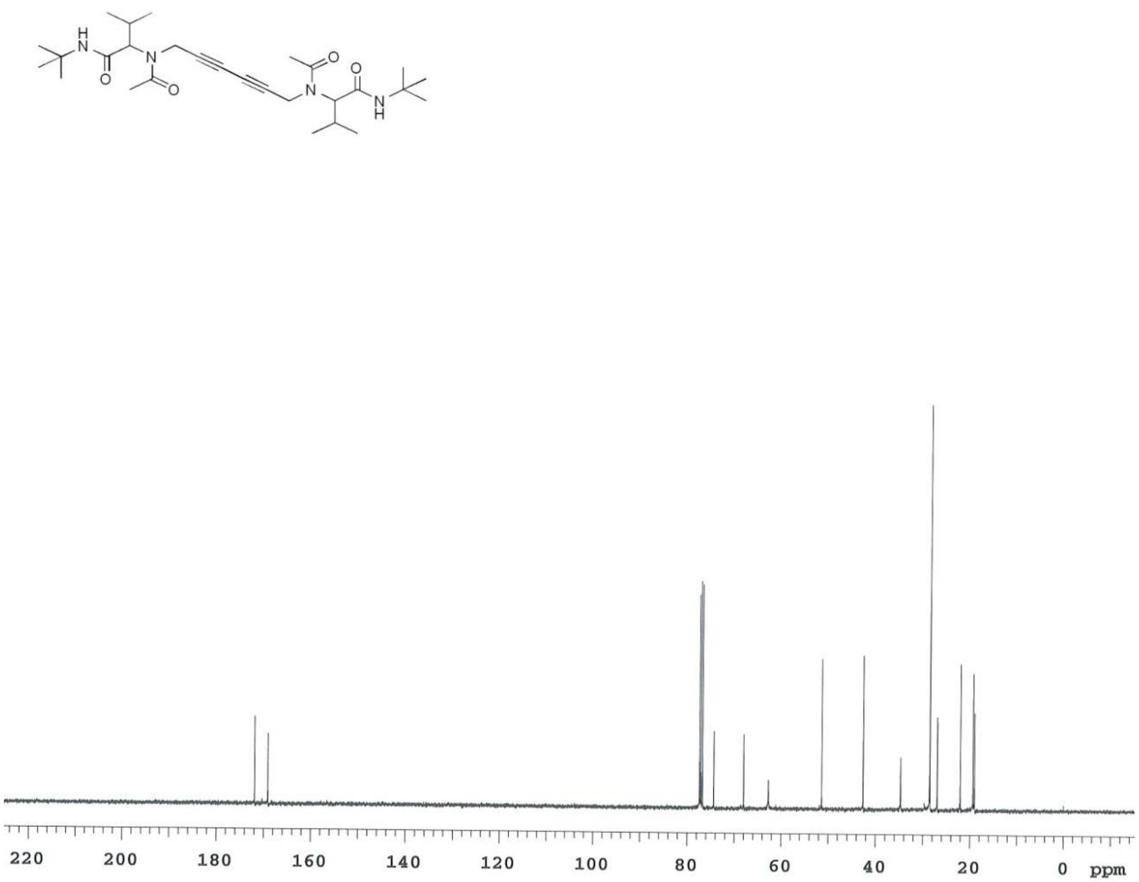

Figure S22: ^{13}C NMR spectrum of compound **8a**

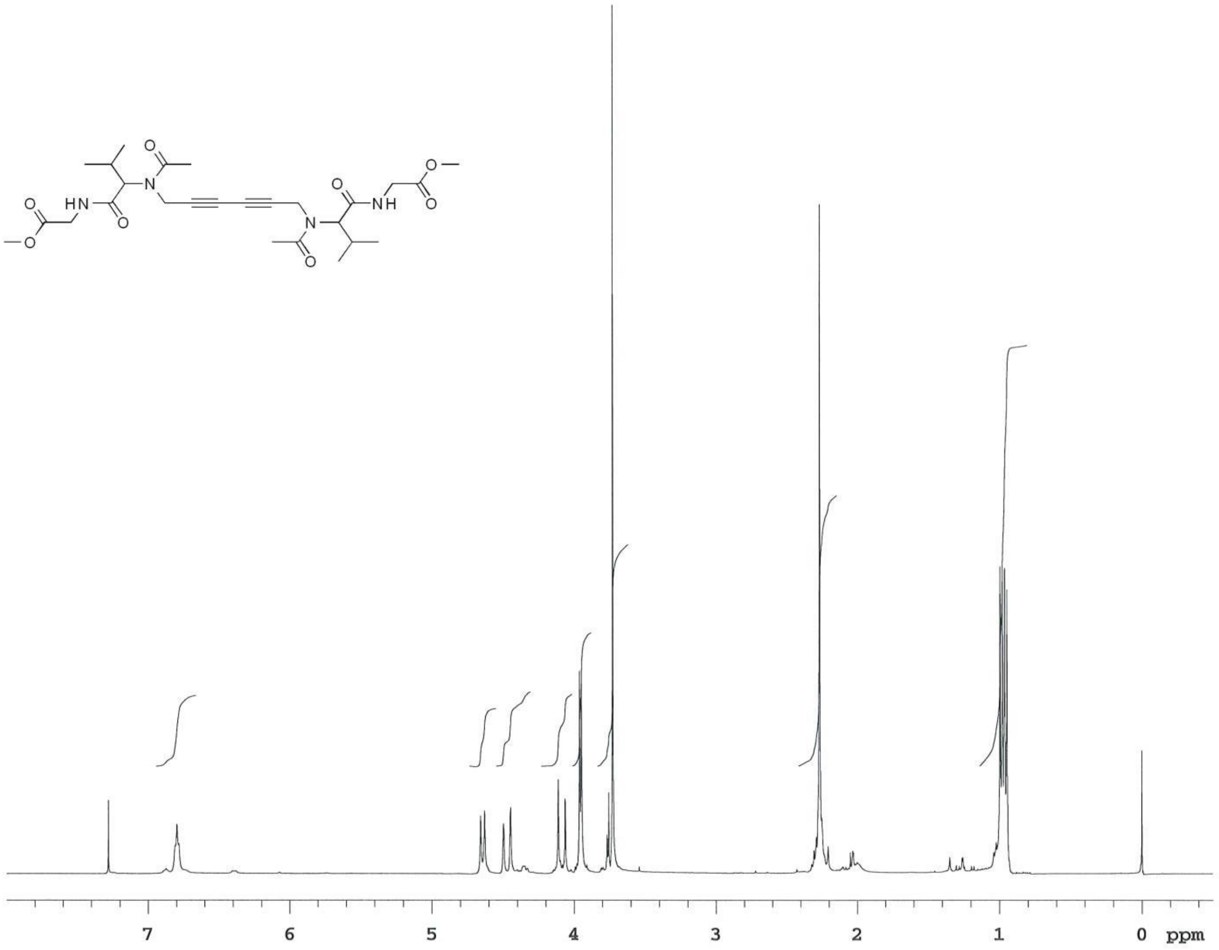

Figure S23: ^{1}H NMR spectrum of compound **8b**

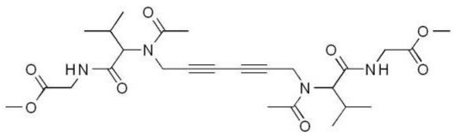

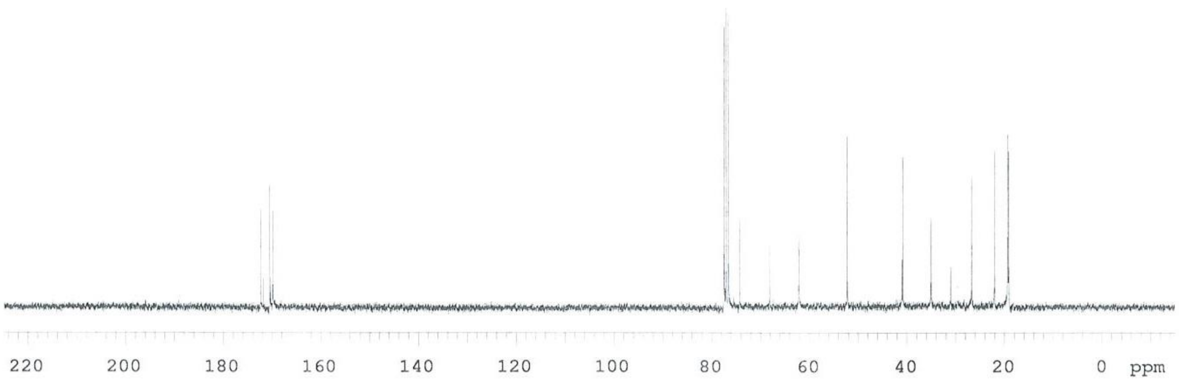

Figure S24: ^{13}C NMR spectrum of compound **8b**

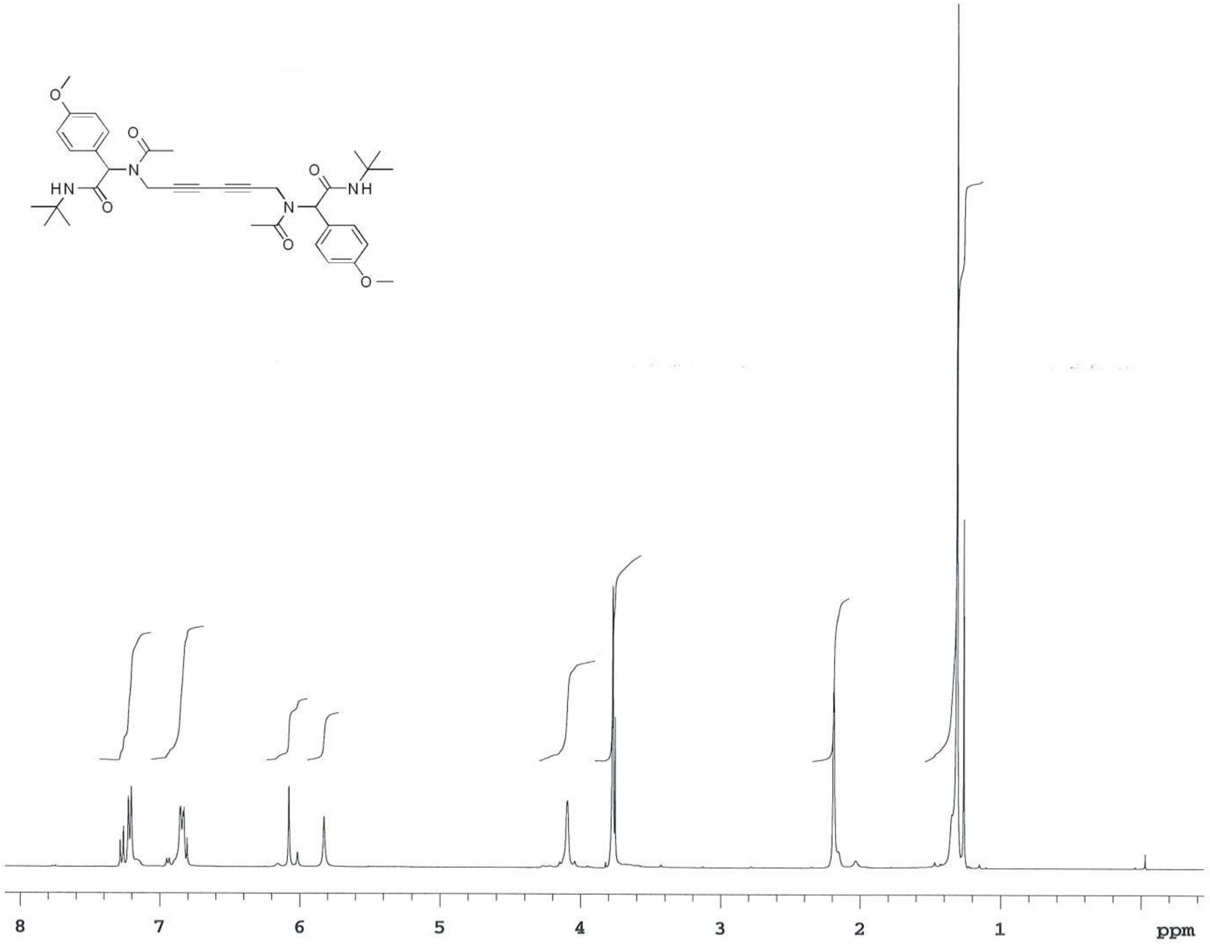

Figure S25: ^{1}H NMR spectrum of compound **8c**

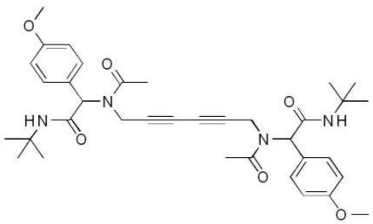

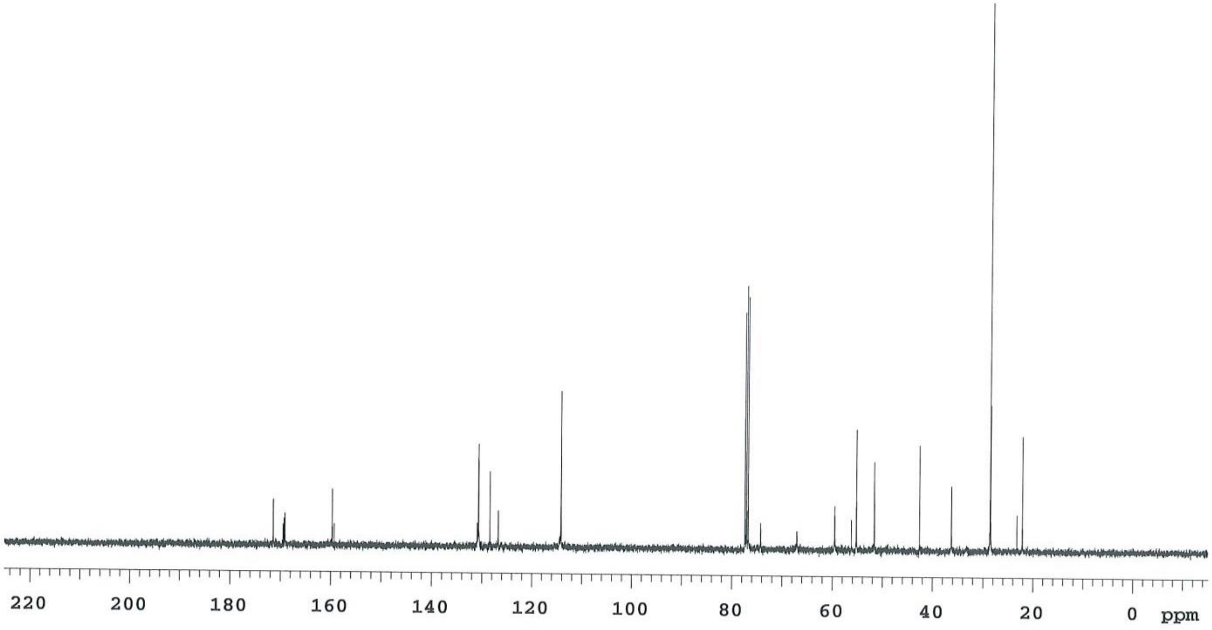

Figure S26: ^{13}C NMR spectrum of compound **8c**

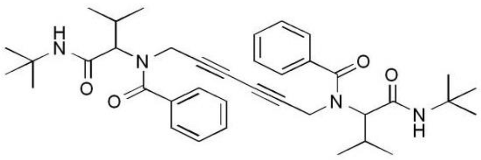

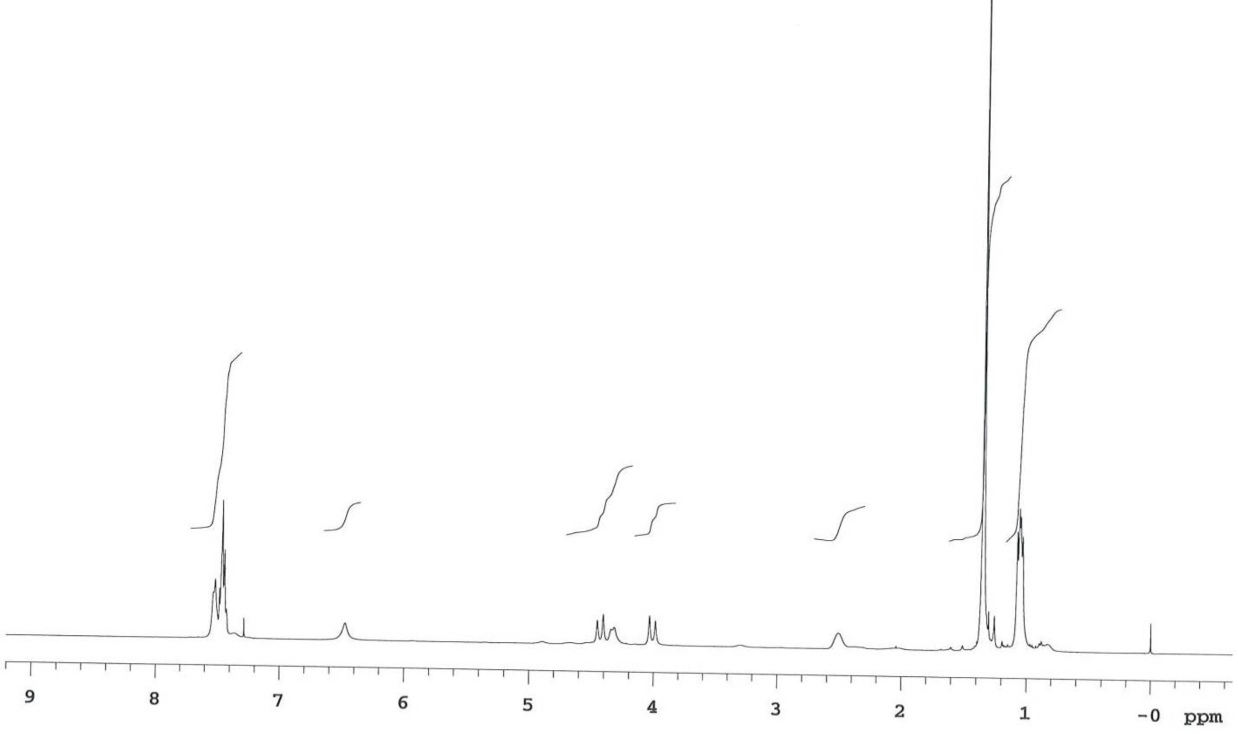

Figure S27: ^{1}H NMR spectrum of compound **8d**

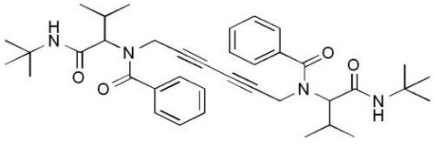

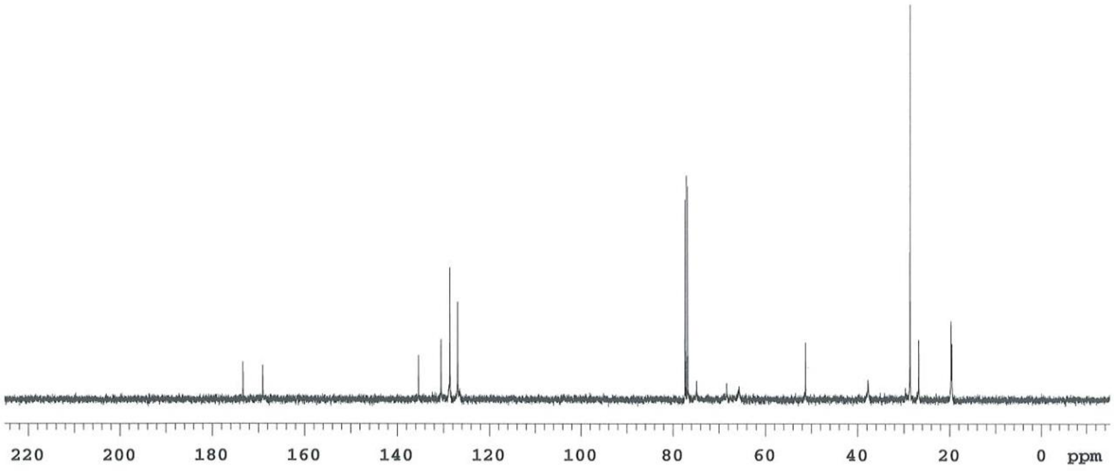

Figure S28: ^{13}C NMR spectrum of compound **8d**

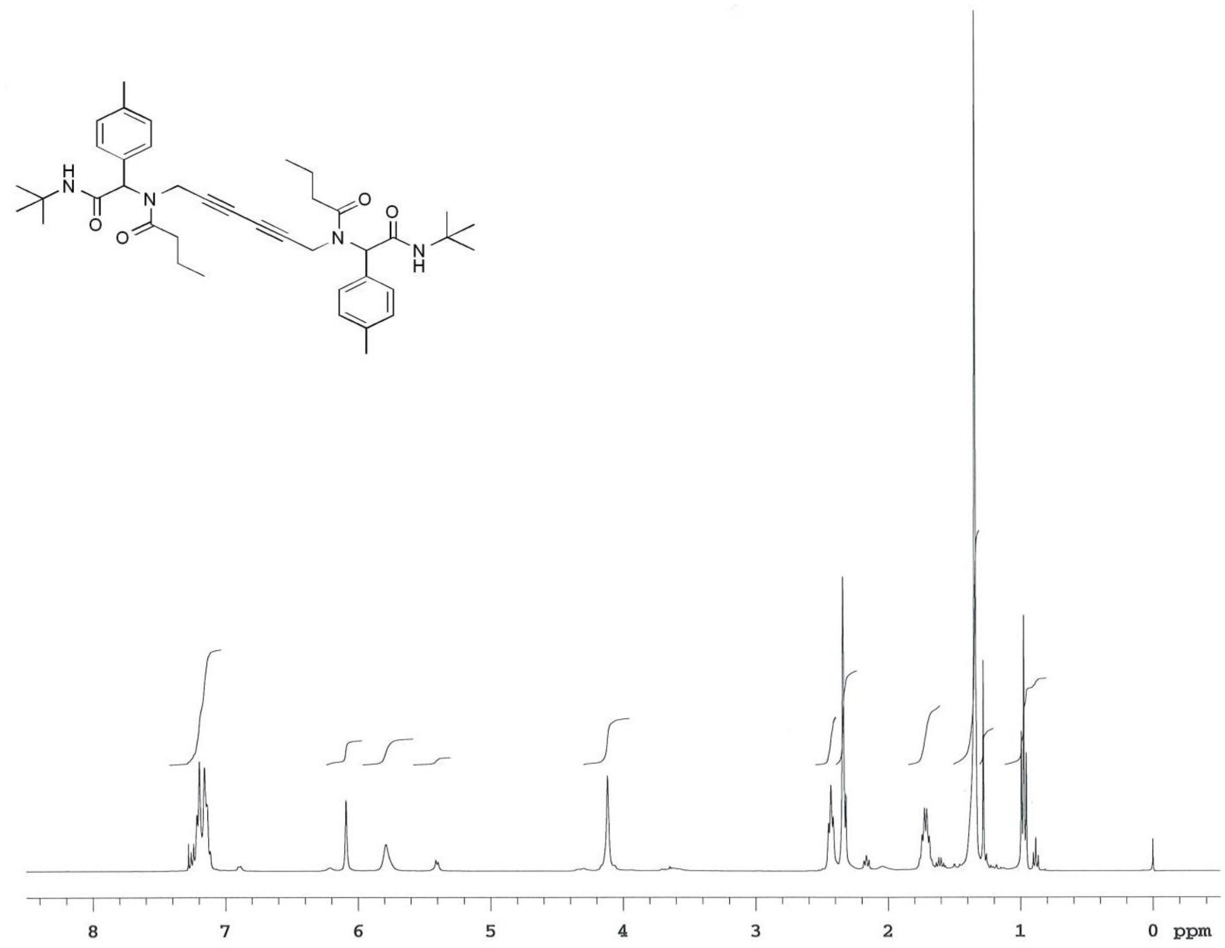

Figure S29: ^{1}H NMR spectrum of compound **8e**

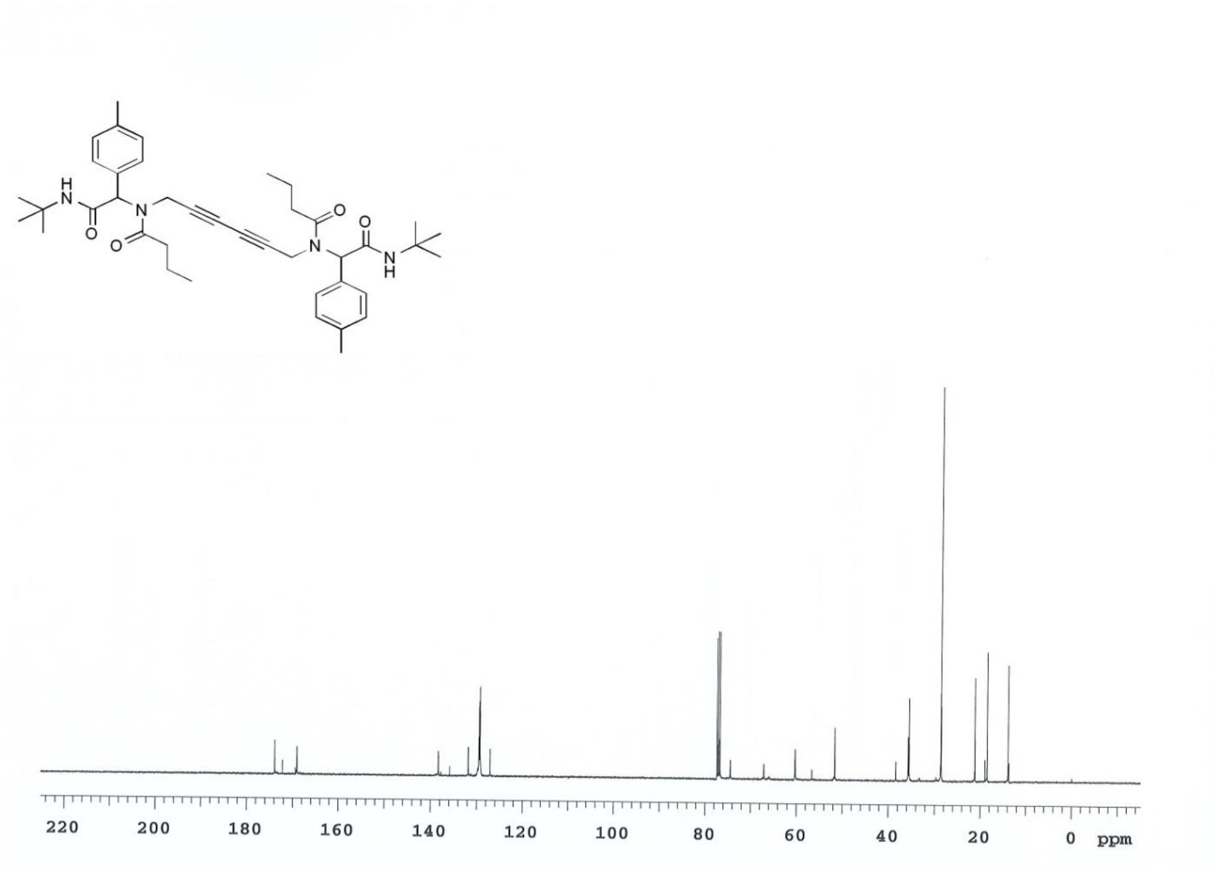

Figure S30: ^{13}C NMR spectrum of compound **8e**

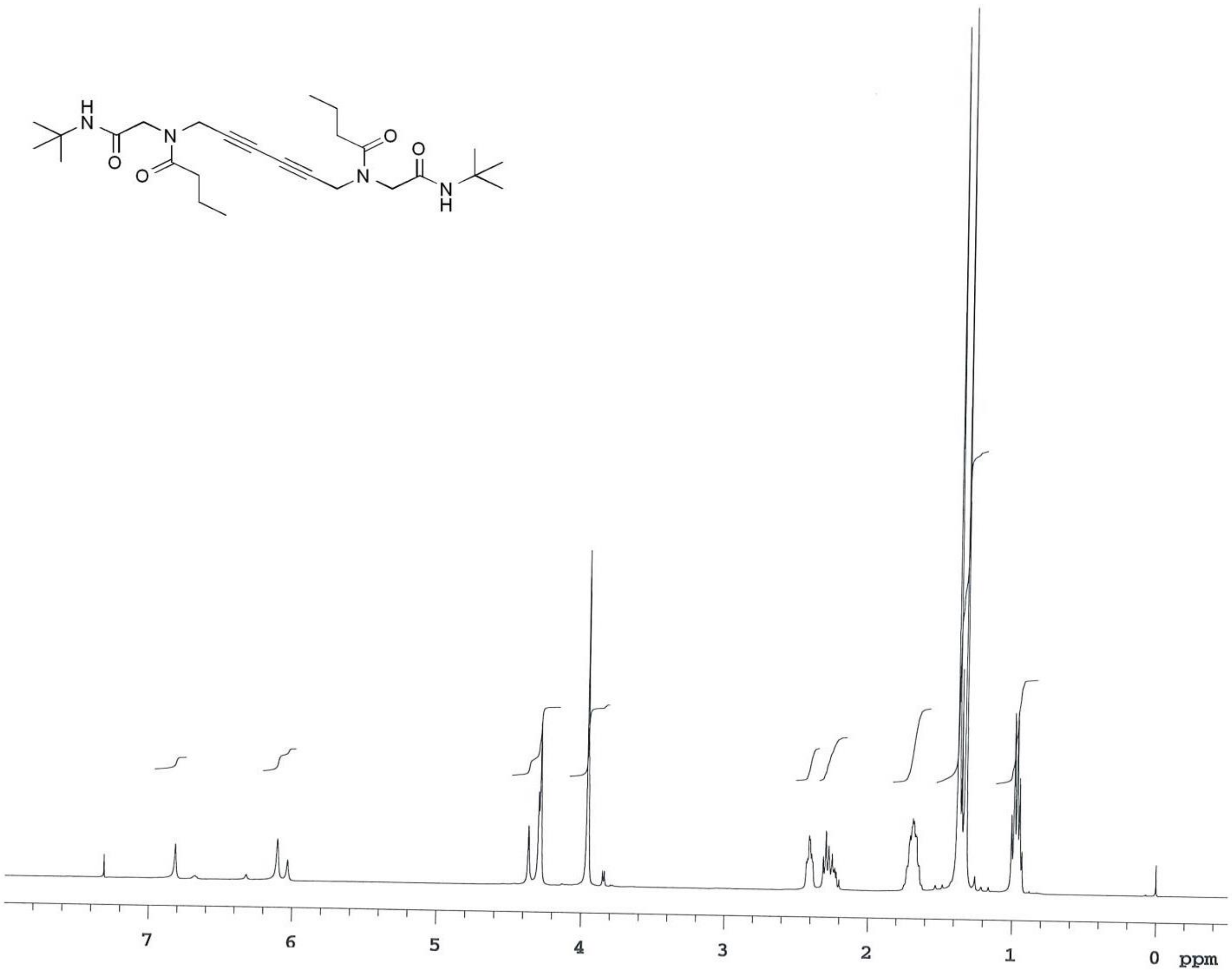

Figure S31: ^{1}H NMR spectrum of compound **8f**

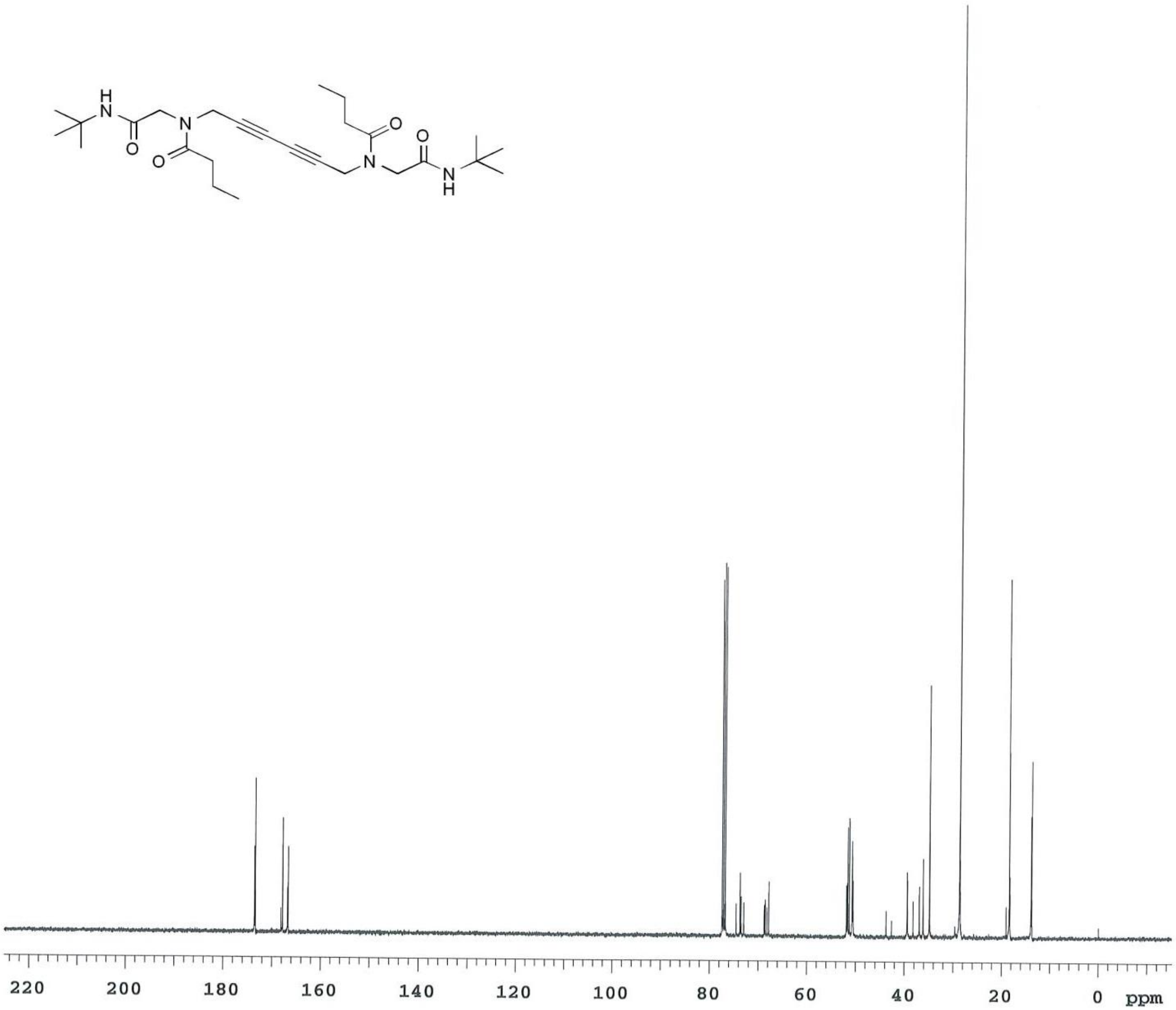

Figure S32: ^{13}C NMR spectrum of compound **8f**

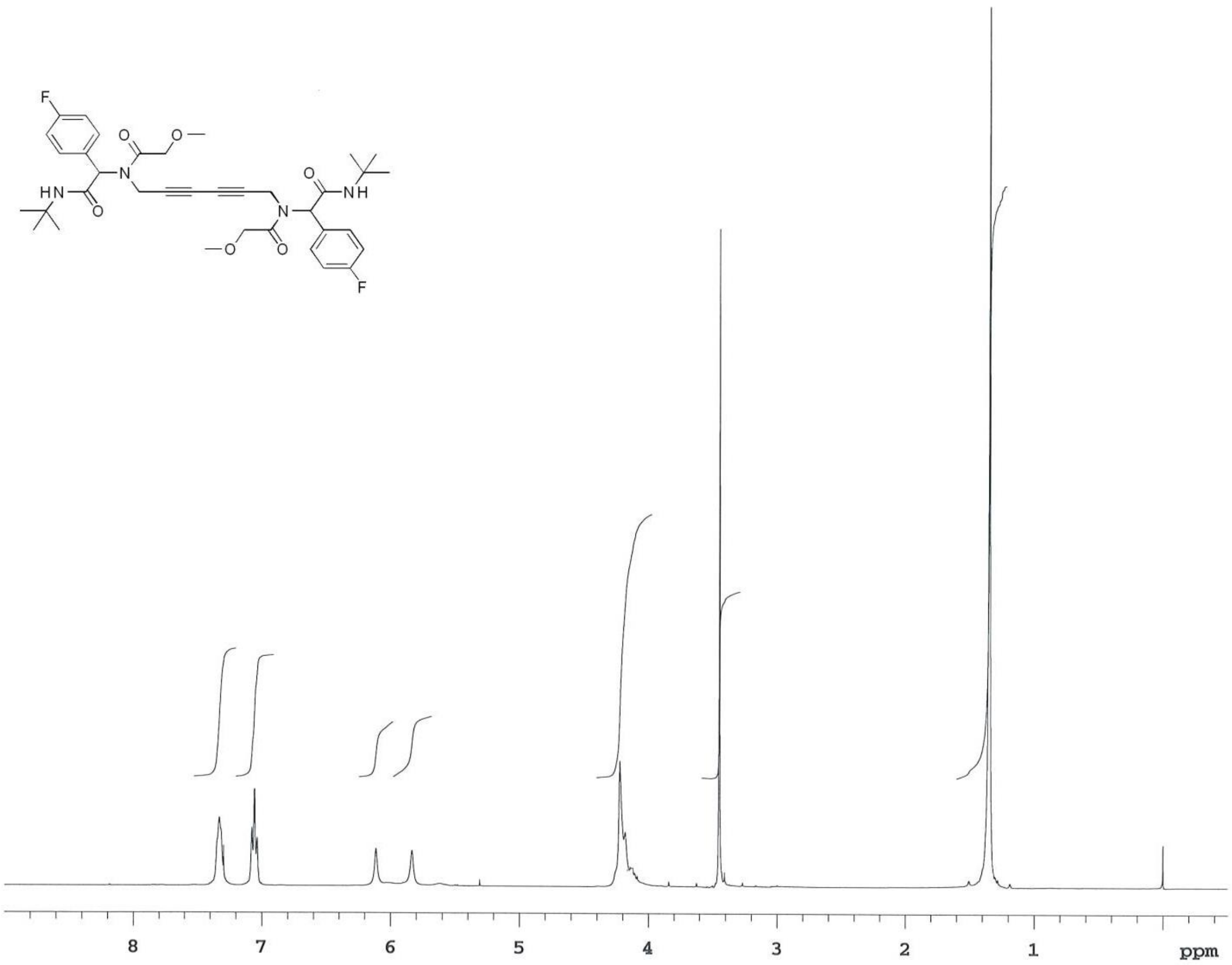

Figure S33: ^{1}H NMR spectrum of compound **8g**

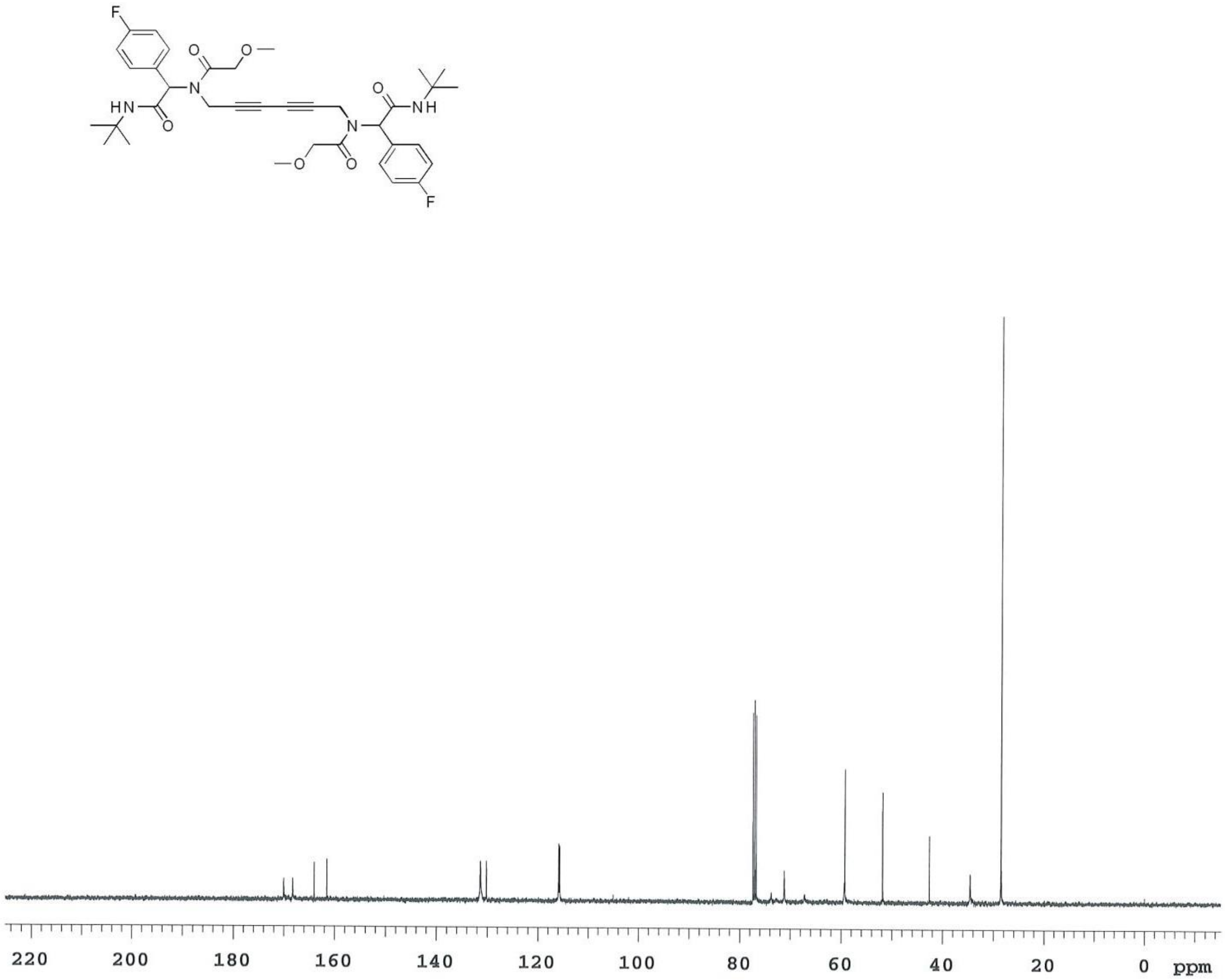

Figure S34: ^{13}C NMR spectrum of compound **8g**

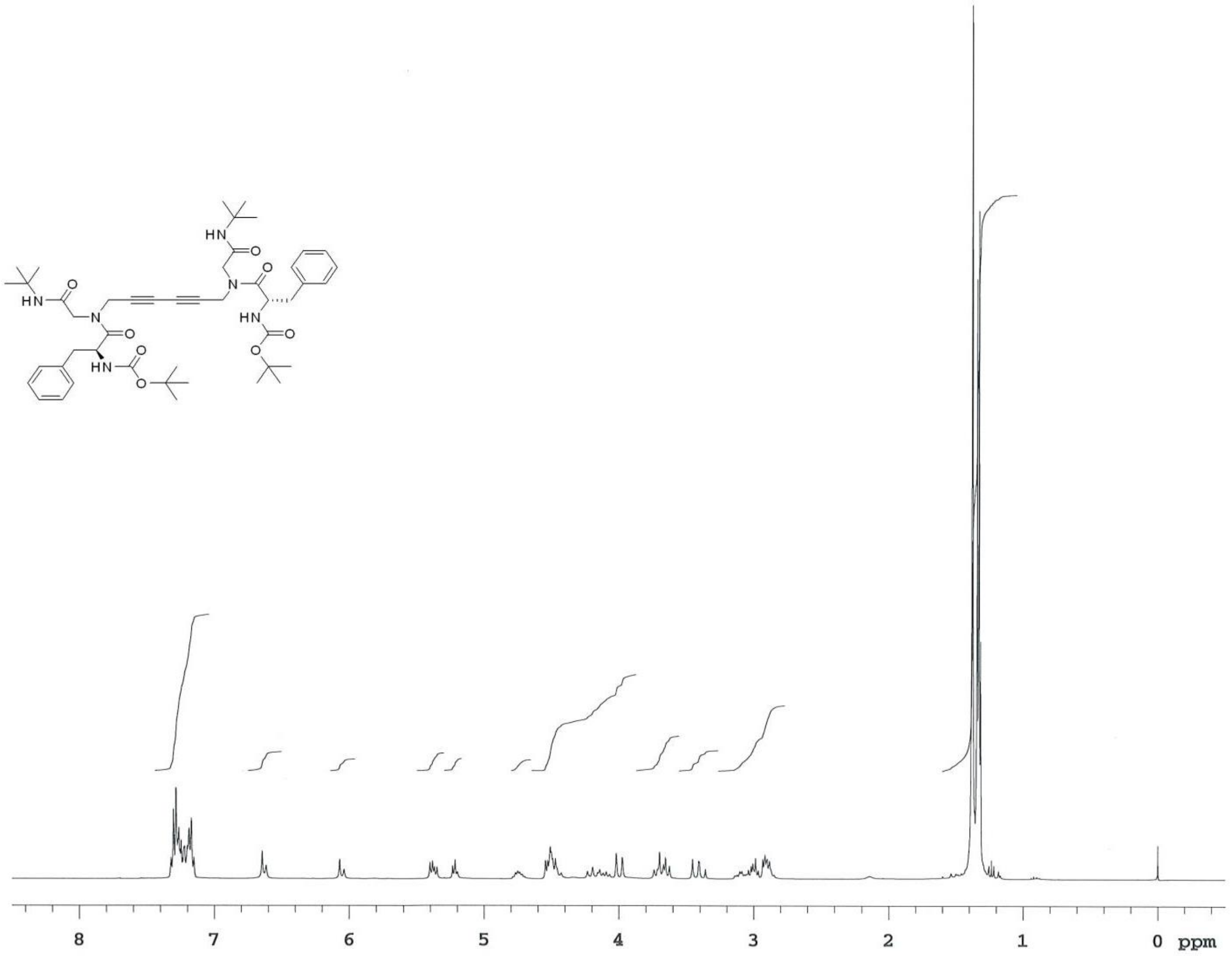

Figure S35: ^{1}H NMR spectrum of compound **8h**

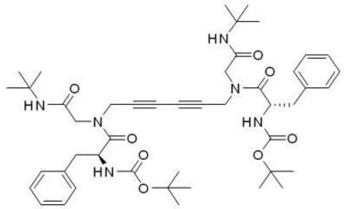

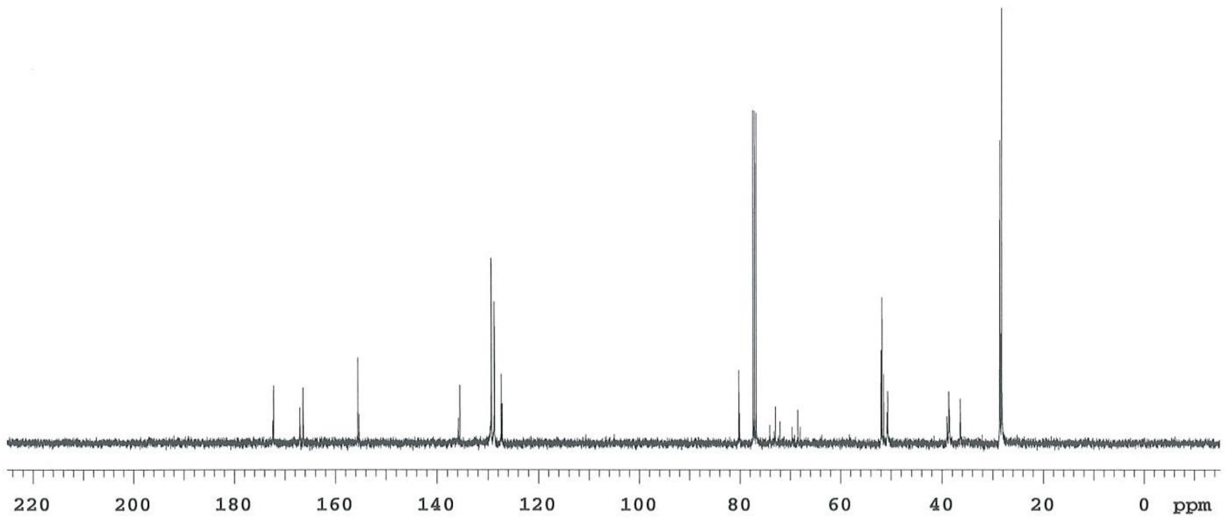

Figure S36: ^{13}C NMR spectrum of compound **8h**

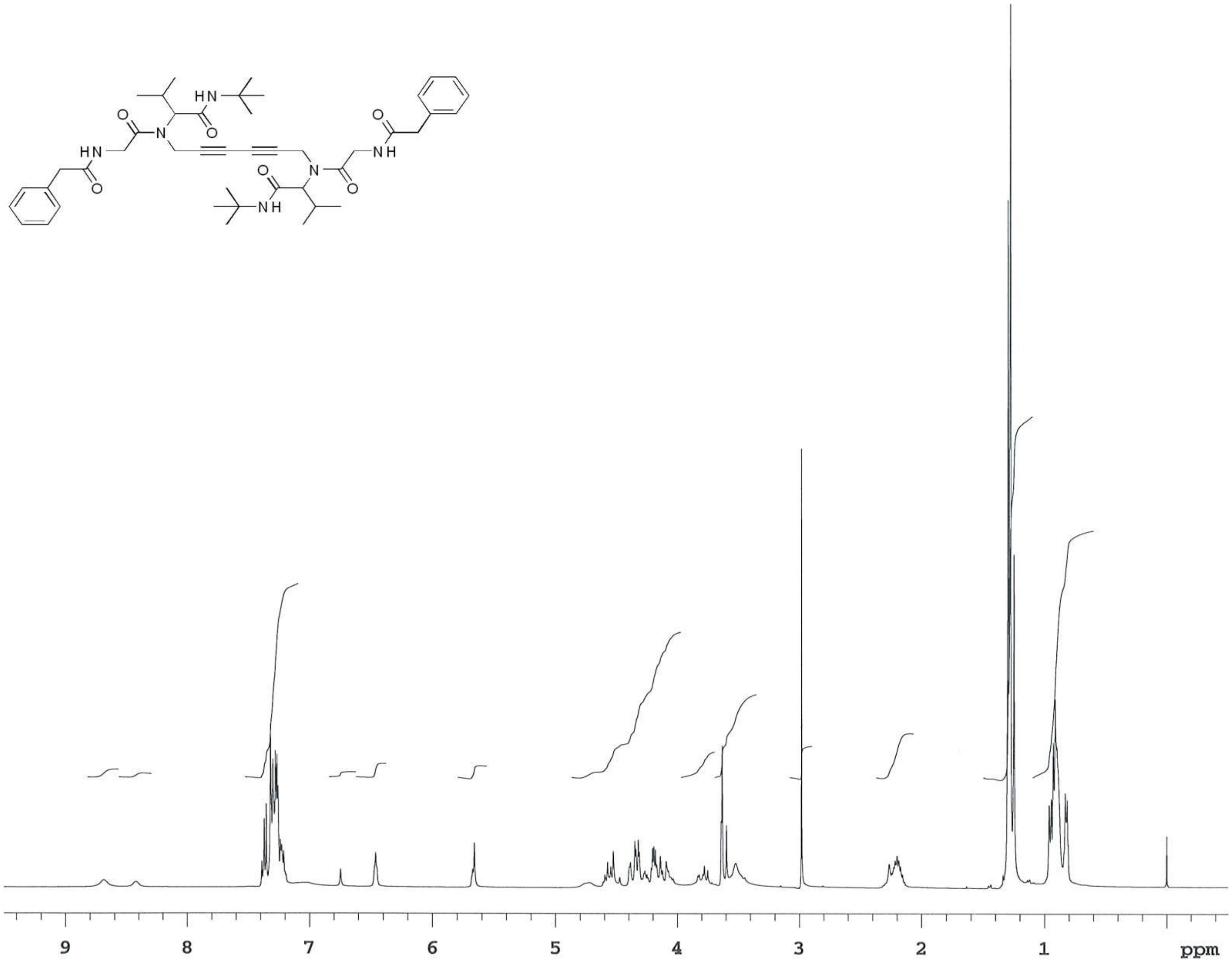

Figure S37: ^{1}H NMR spectrum of compound **8i**

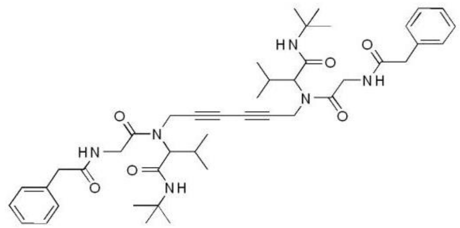

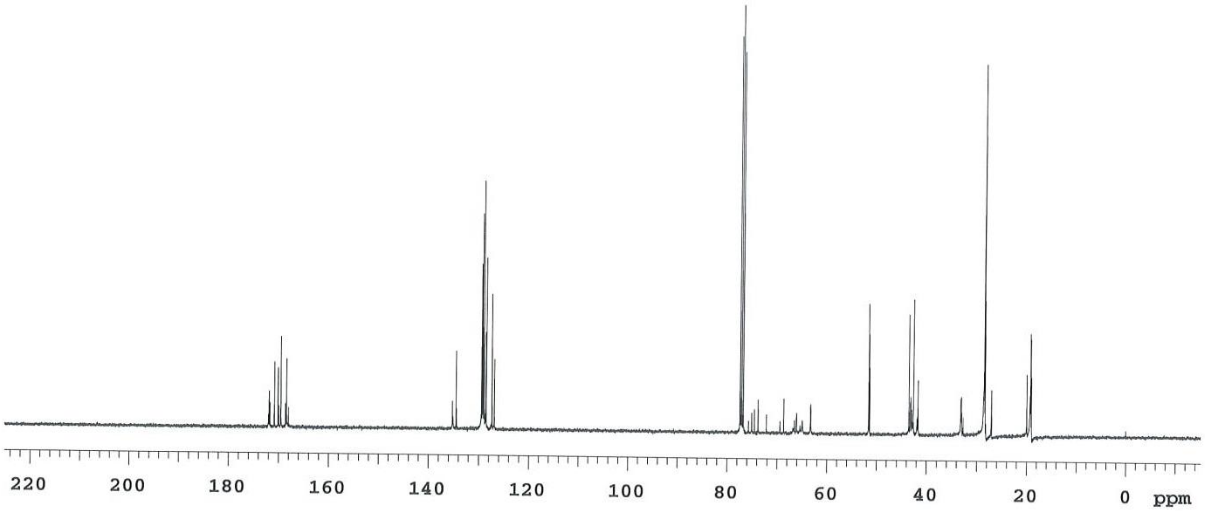

Figure S38: ^{13}C NMR spectrum of compound **8i**

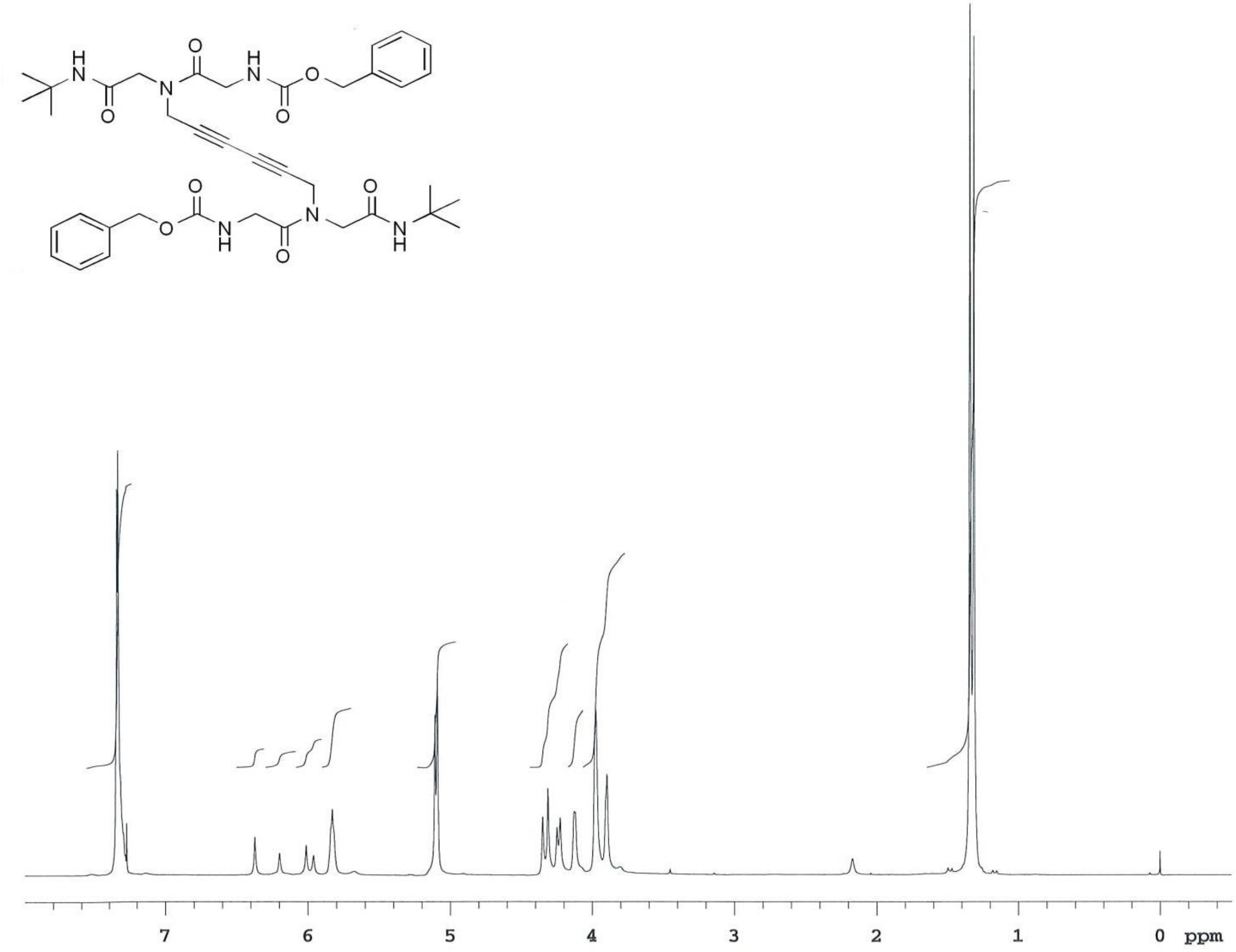

Figure S39: ^{1}H NMR spectrum of compound **8j**

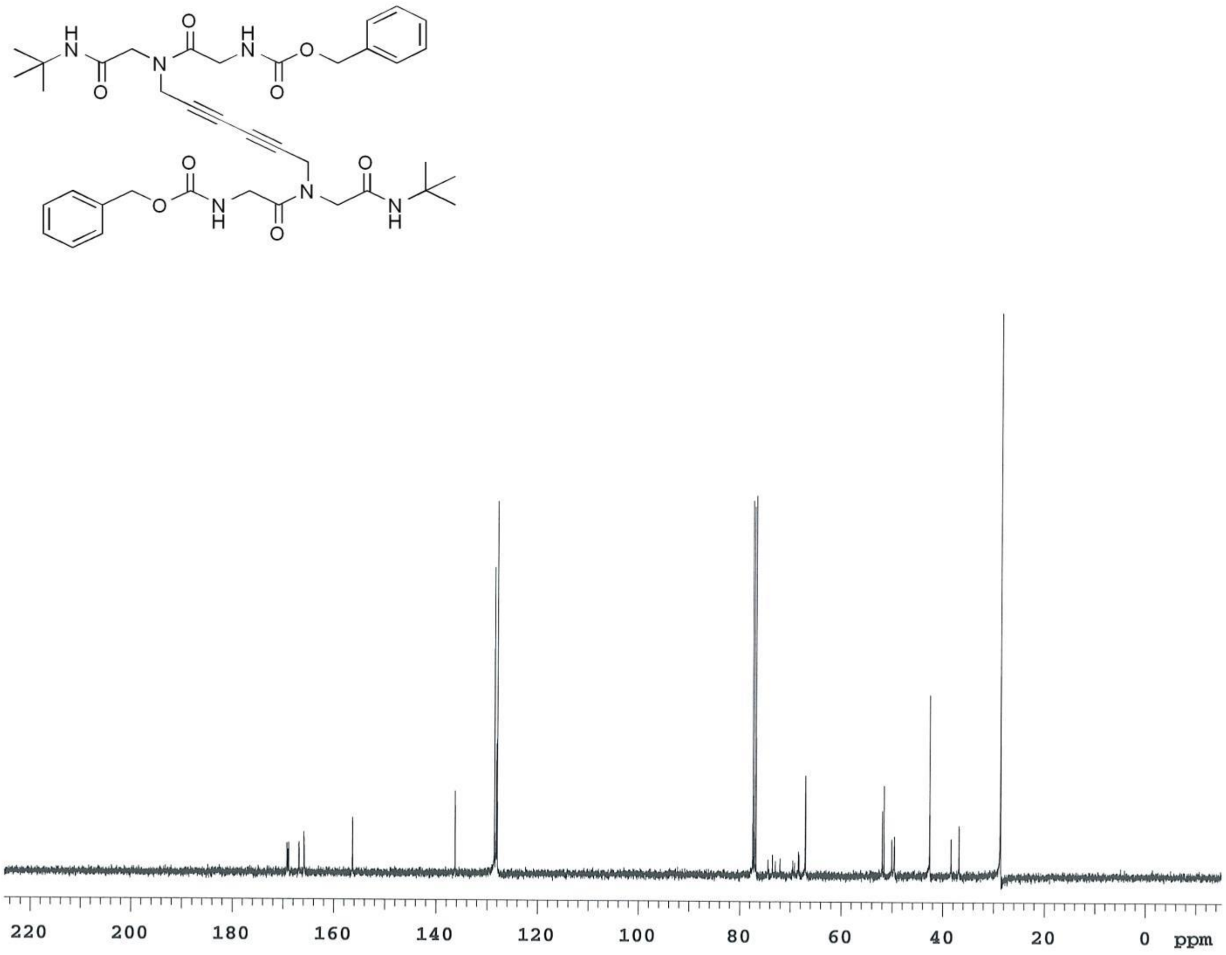

Figure S40: ^{13}C NMR spectrum of compound **8j**
